# A Structurally Specialized Uniform Wall Layer is Essential for Constructing Wall Ingrowth Papillae in Transfer Cells

**DOI:** 10.3389/fpls.2017.02035

**Published:** 2017-12-05

**Authors:** Xue Xia, Hui-Ming Zhang, Christina E. Offler, John W. Patrick

**Affiliations:** School of Environmental and Life Sciences, University of Newcastle, Callaghan, NSW, Australia

**Keywords:** cellulose microfibril, cellulose synthase, cortical microtubule array, seed, transfer cell, wall ingrowths

## Abstract

Transfer cells are characterized by wall labyrinths with either a flange or reticulate architecture. A literature survey established that reticulate wall ingrowth papillae ubiquitously arise from a modified component of their wall labyrinth, termed the uniform wall layer; a structure absent from flange transfer cells. This finding sparked an investigation of the deposition characteristics and role of the uniform wall layer using a *Vicia faba* cotyledon culture system. On transfer of cotyledons to culture, their adaxial epidermal cells spontaneously *trans*-differentiate to a reticulate architecture comparable to their abaxial epidermal transfer cell counterparts formed *in planta*. Uniform wall layer construction commenced once adaxial epidermal cell expansion had ceased to overlay the original outer periclinal wall on its inner surface. In contrast to the dense ring-like lattice of cellulose microfibrils in the original primary wall, the uniform wall layer was characterized by a sparsely dispersed array of linear cellulose microfibrils. A re-modeled cortical microtubule array exerted no influence on uniform wall layer formation or on its cellulose microfibril organization. Surprisingly, formation of the uniform wall layer was not dependent upon depositing a cellulose scaffold. In contrast, uniform wall cellulose microfibrils were essential precursors for constructing wall ingrowth papillae. On converging to form wall ingrowth papillae, the cellulose microfibril diameters increased 3-fold. This event correlated with up-regulated differential, and transfer-cell specific, expression of *VfCesA3B* while transcript levels of other cellulose biosynthetic-related genes linked with primary wall construction were substantially down-regulated.

## Introduction

Transfer cells (TCs) are located at key sites throughout the plant body where they support high transport fluxes of nutrients between apo- and symplasmic compartments. Broadly these sites function in nutrient acquisition across interfaces including soil/root, maternal/filial tissues of developing seeds and host/biotroph or in loading/unloading of vascular pipelines to regulate nutrient partitioning between competing sinks (Pate and Gunning, 1972; Offler et al., 2003; Andriunas et al., 2013). Membrane transport capacities of TCs are proportionate to the amplification of their transporter-enriched plasma membrane surface areas (up to 20-fold). Inturn the magnitude of membrane amplification is determined by the degree of invagination of the TC wall labyrinth on which the amplified plasma membrane is arrayed. The structural organization of the wall labyrinth can be one of three architectural designs—reticulate, flange or reticulate ingrowths deposited on flanges (Pate and Gunning, 1972; Andriunas et al., 2013).

Of the types of wall labyrinths exhibited by TCs, the reticulate design provides the greatest plasticity for amplifying plasma membrane surface areas. The degree of reticulation has been found to range from a single layer of discrete or branched wall ingrowth (WI) papillae to repeating fenestrated layers of WI papillae that branch and fuse (Pate and Gunning, 1972; Andriunas et al., 2013). The selective advantage of the reticulate wall labyrinth design is illustrated by its occurrence in species of all plant taxa from algae to angiosperms (Offler et al., 2003).

**Figure 1:**
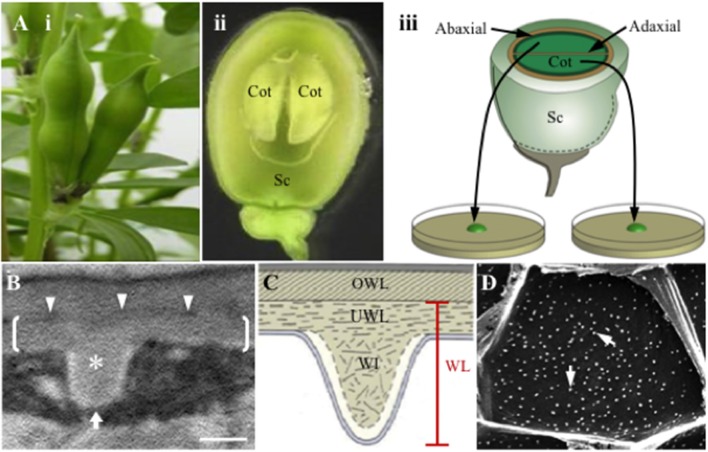
Schematic of the *Vicia faba* cotyledon culture system and illustrations of the wall labyrinth forming in *trans*-differentiating adaxial epidermal transfer cells of cultured cotyledons. **(A)** Schematic of the *Vicia faba* cotyledon culture system illustrating (i) pods prior to harvest, (ii) a seed, cut in half longitudinally, showing the coat (Sc) enclosing two “sister” cotyledons (Cot), (iii) “sister” cotyledons removed from the seed coat, are separated and placed adaxial epidermal surface down on the culture medium. **(B,C)** Transmission electron micrograph **(B)** and diagrammatic representation **(C)** of a transverse section of the outer periclinal wall of a *trans*-differentiating adaxial epidermal transfer cell illustrating the structure of the wall labyrinth (WL). Brackets in **(B)** mark the uniform wall layer (UWL) deposited on the original wall (OWL) and demarcated by an electron-dense band (darts in **B**); a wall ingrowth (WI) papilla is marked with an asterisk in **(B)**. **(D)** Scanning electron micrograph of WI papillae deposition at the cytoplasmic face of the outer periclinal wall of adaxial epidermal cells. Note each of the white dots represent a single WI papilla (arrows). The white arrow in **(B)** indicates the direction of imaging of the cytoplasmic face of the outer periclinal wall. Bar, 300 nm in **(B)** and 10 μm in **(D)**.

Given the role TCs play in determining capacity for membrane transport, most attention has been paid to the composition and construction of WI papillae of reticulate wall labyrinths. Progress in understanding these phenomena has been facilitated by the development of a TC inductive system (Figure 1A), whereby adaxial epidermal cells of developing *Vicia faba* cotyledons, on being transferred to culture, undergo spontaneous *trans*-differentiation to an epidermal transfer cell (ETC) morphology and function comparable to their abaxial counterparts (Offler et al., 1997; Farley et al., 2000). Studies using this experimental system have discovered that fully-developed WI papillae are comprised of a core of cellulose microfibrils arranged in whorls at right angles to the underlying wall. The cellulose core of each WI papilla is encased by an electron translucent sheath (Figures 1B,C), possibly containing callose, but devoid of cellulose and other matrix wall materials (Talbot et al., 2007; Vaughn et al., 2007). WI papillae construction commences at 3 h of cotyledon culture and their initiation is completed by 15 h (Wardini et al., 2007). Persistent plumes of elevated cytosolic Ca^2+^ define loci at which WI papillae form (Zhang et al., 2015a). An ongoing deposition of cellulose provides an essential scaffold for their assembly (Talbot et al., 2007). While re-modeling of the cortical microtubule network co-occurs with WI papillae deposition, the network does not play any role in directing their construction (Zhang et al., 2015b).

For ETCs of *V. faba* cotyledons, WI papillae arise from a distinctive and polarized wall layer deposited inside the outer periclinal region of the original wall (Figures 1B,C, also see Offler et al., 1997; Farley et al., 2000) termed the uniform wall layer (McCurdy et al., 2008). Together the uniform wall layer and WI papillae form the reticulate wall labyrinth that, based on an immunological study (Vaughn et al., 2007), has a polysaccharide composition consistent with a primary cell wall. A literature survey shows that the uniform wall layer, identified by a thickened wall from which the WI papillae arise, is a ubiquitous structural feature of reticulate wall labyrinths located in species of all plant taxonomic groups but is absent from flange wall labyrinths (Supplementary Table S1). Current knowledge of uniform wall layer construction in t*rans*-differentiating epidermal cells of *V. faba* cotyledons is that it is regulated by extracellular reactive oxygen species (ROS; Andriunas et al., 2012; Xia et al., 2012), precedes WI papillae deposition and reaches completion by 10 h of cotyledon culture (Zhang et al., 2015c).

Given that WI papillae formation is cellulose dependent (see Figure 4 in Talbot et al., 2007), we explored the hypothesis that cellulose deposition plays a similar role in constructing the uniform wall layer and could contribute to the subsequent assembly of WI papillae. Visualization of cellulose microbrils in cell walls demonstrated that their deposition patterns differed substantially between the original primary wall, the uniform wall layer and WI papillae. Similar to assembly of WI papillae (Zhang et al., 2015b), cellulose microfibril deposition in the uniform wall layer was found to be microtubule independent. However, in contrast to WI papillae formation (Talbot et al., 2007), pharmacological blockade of cellulose biosynthesis did not compromise the volume of uniform wall layer formed. Rather uniform wall layer cellulose was shown to serve as an essential precursor for WI papillae assembly with diameters of these precursor cellulose microfibrils increasing 3-fold. A temporal expression analysis of genes encoding cellulose-related enzymes across the sequential construction phases of the original primary wall, uniform wall layer and WI papillae, found that up-regulated expression of a *VfCesA3* isoform coincided with the transition from uniform wall layer to WI papillae assembly.

## Materials and methods

### Plant growth and cotyledon culture conditions

Developing seeds were harvested from *V. faba* L. (cv. Fiord) plants raised under controlled environmental conditions. Cotyledons were surgically removed from their seed coats and prepared for aseptic culture on a Murashige and Skoog (MS) medium (Murashige and Skoog, 1962) ± specified pharmacological reagents as previously described (Zhou et al., 2010).

### Visualizing wall labyrinth structure by transmission and scanning electron microscopy

Temporal changes in the deposition pattern and dimensions of the original and uniform wall layer were determined from 65 nm transverse ultrathin sections of adaxial epidermal cells of cultured cotyledons (Figure 1A). Harvested cotyledons were cut into several pyramid shaped pieces with a 2 × 3 mm base of adaxial epidermal cells. Tissue blocks were then fixed and dehydrated for preparation for transmission electron microscopy (TEM) following protocols outlined by Farley et al. (2000) and visualized using a JEOL 12000 EX II transmission electron microscope (JEOL, Japan).

For visualization of the WI papillae deposited in the outer periclinal walls of adaxial epidermal cells of cultured cotyledons by scanning electron microscopy (SEM), strips of epidermal cells (average size 15 mm^2^ after critical point drying) were peeled from the cultured cotyledons. The peels, with many cells fractured at their anticlinal walls (Dibley et al., 2009), were then cleared in 2% NaOCl for 3 h to remove the protoplasm and sequentially dehydrated and critical point dried following the protocol detailed in Zhou et al. (2010). The outer face of the outer periclinal walls of peels was then stuck on aluminum stubs using carbon paint allowing their cytoplasmic face to be observed (Figure 1D) using a Philips XL30 scanning electron microscope (SEM) to record WI papillae formation as described by Zhou et al. (2010).

### Visualizing cellulose microfibrils of the outer periclinal epidermal cell wall by field emission scanning electron microscopy

Tissue preparation for field emission scanning electron microscopy (FESEM) was based on an acid hydrolysis procedure developed by Sugimoto et al. (2000) that removes wall components other than crystalline cellulose microfibrils. We adapted this procedure for clearing the delicate epidermal peels fractured at their anticlinal walls. The epidermal peels were immediately boiled in acetic acid: nitric acid: dH_2_O (8:1:2) for various lengths of time to remove their protoplasts and cell wall matrix polysaccharides including hemicellulose (Sugimoto et al., 2000) leaving the cellulose microfibril superstructure. After 3 × 10-min rinses in dH_2_O, the “cleared” epidermal peels were dehydrated through 10% ethanol steps to 100% ethanol at 30-min intervals. Following air-drying to minimize their curling (see Carpita et al., 2001), the peels were stuck, adaxial surface down, on aluminum stubs using carbon paint. The mounted peels were sputter coated with platinum at 10 mA for 30 s to result in a platinum coating thickness of 3 nm as specified by the manufacturer (SPI Suppliers, USA). The layers of cellulose microfibrils in the outer periclinal cell walls were imaged using a Zeiss VP FESEM at 2 kV fitted with a ZEISS Gemini ® in-lens detector with a capability of gathering electron interactions up to a depth of 500 nm into the sample. Thus, electrons penetrating through spaces between cellulose microfibrils allowed layers of cellulose microfibrils to be imaged. High-resolution micrographs were taken with a working distance of 3 mm. Objective apertures were set at 20 μm and, unless specified otherwise, at a contrast setting of 43%.

**Figure 2:**
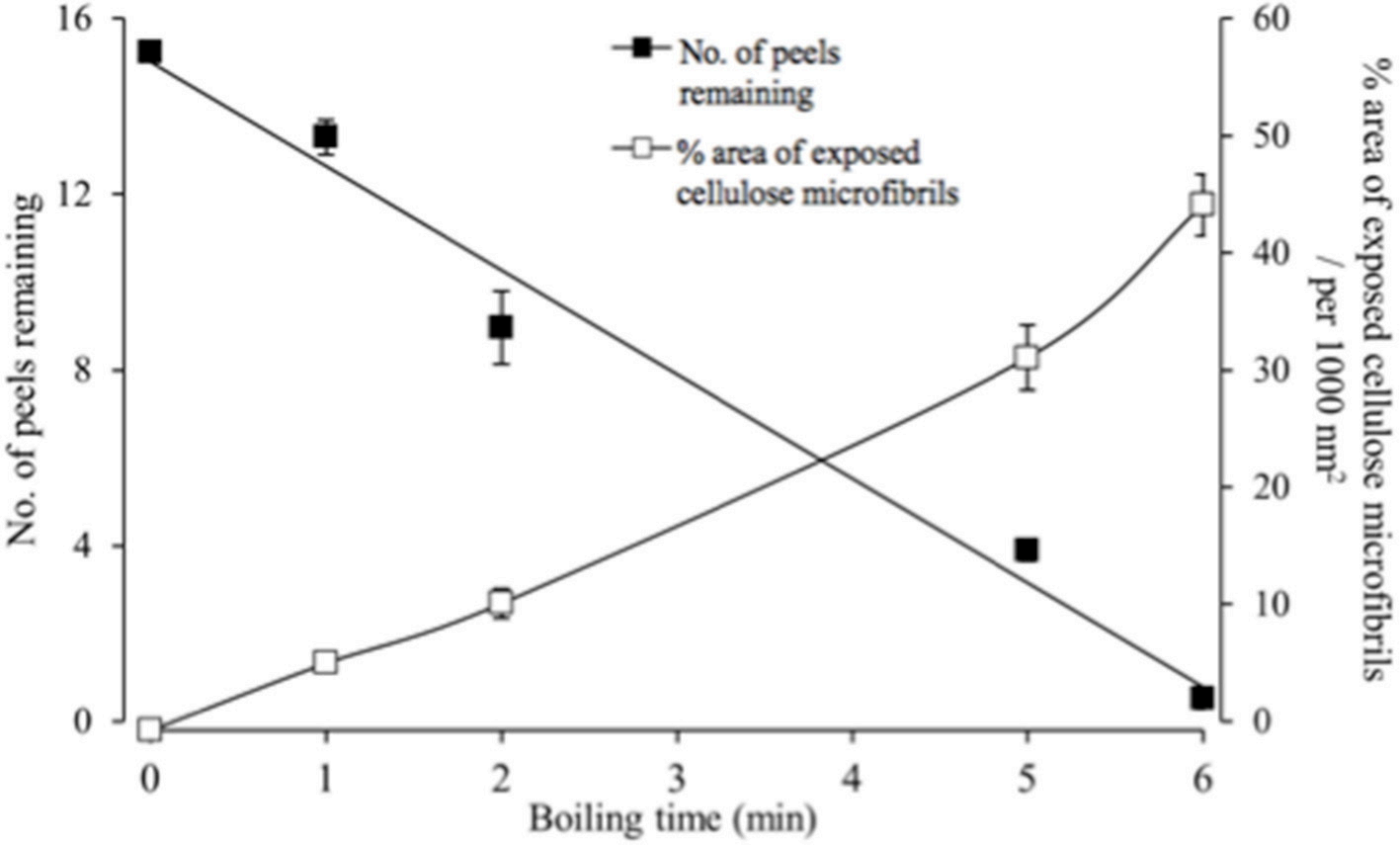
Temporal impact of acid hydrolysis on the number of fractured epidermal peels and their relative areas of exposed cellulose microfibrils visualized by FESEM. For each peel, 20 randomly selected fields of view were assessed to determine the % area of exposed cellulose microfibrils.

The epidermal peels were rapidly hydrolysed in the acid bath. Hence a balance was sought between optimizing areas of exposed cellulose microfibrils available for FESEM observation and remaining peel numbers to permit statistical estimates of cellulose microfibril properties. A 5-min acid hydrolysis best met these criteria starting with a batch of 16 peels, with one peel per replicate cotyledon (Figure 2).

### Measures of uniform wall layer cellulose microfibrils

#### Percent cell wall coverage by cellulose microfibrils

Cells of cleared epidermal peels (see above) were imaged by FESEM at 100 kx magnification. In randomly chosen fields of view (3.1 × 2.1 μm), in which the cellulose microfibrils of the original wall were apparent, presence/ absence of uniform wall layer cellulose microfibrils was recorded (see Supplementary Figure S1). Where the field of view was only partially occupied by a cellulose patch, a relative estimate of this coverage was made. Fifteen to 20 observations were recorded per cell across five cells per cotyledon with four replicates per treatment. The number of observations in which uniform wall layer cellulose microfibrils were detected was expressed as a percentage of the total observations to provide a relative measure of uniform wall layer cellulose microfibril coverage per cell.

#### Relative densities of cellulose microfibrils

Images were captured of those fields of view exhibiting original and uniform wall layer cellulose microfibrils (see above). Each image was overlain by a randomly-positioned grid comprised of 4 × 4 squares (each 600 nm). Numbers of intersections by cellulose microfibrils across the grid lines were scored to provide a relative estimate of their densities. This was repeated 5 times per image and then replicated as described above for estimates of cellulose microfibril coverage.

#### Diameters of cellulose microfibrils

Image J length selection tool was used to measure diameters of cellulose microfibrils captured in FESEM images of the “cleared” original or uniform wall layers corrected for the thickness of the platinum coating. The thickness of sputtered platinum coating was estimated to be 3 nm according to the manufacturer's instructions (see https://mc2.engin.umich.edu/wp-content/uploads/sites/227/2015/11/sputter_coater.pdf) for being applied in an argon atmosphere at a plasma current of 9 mA and applied voltage of 1 kV for 30 s.

### Original and uniform wall layer dimensions of adaxial epidermal cells

#### Cell and cotyledon measurements

Transmission electron micrographs of transverse sections of each adaxial epidermal cell of cultured cotyledons were scanned with the Image J freehand selection tool to obtain measures of the cross-sectional areas of their original and uniform wall layers along with their respective widths (see Supplementary Table S2). Ratios of transverse area to width measures (μm^2^/μm) provided averaged estimates of wall layer thickness. Measures of cell lengths and outer periclinal surface areas were obtained from SEM and confocal laser scanning micrographs of epidermal peels using Image J software (https://imagej.nih.gov/ij/, see Supplementary Table S2). To express the cell wall dimensions on a cotyledon basis (see below), adaxial surfaces of cultured cotyledons were measured from scans of their digital images using Image J (see Supplementary Table S2).

#### Cell wall volume estimates per cotyledon

Estimates of the original and uniform wall layer volumes per adaxial epidermal cell were derived from the product of their mean cross-sectional areas per cell summed over mean epidermal cell lengths at specified times of cotyledon culture (see Supplementary Table S2).

To estimate wall layer volumes per cotyledon, the derived cell wall volumes (see above) were summed over the total cell numbers per cotyledon. The latter values were deduced from the ratios of cotyledon to cell surface areas (see Supplementary Table S2). In the case of the uniform wall layer volumes per cotyledon, these estimates were calibrated to the percentage of cells exhibiting an uniform wall layer (Supplementary Table S2).

### RNA isolation and cDNA synthesis

Extraction of total RNA from epidermal peels and tissue segments of underlying storage parenchyma of cultured cotyledons and subsequent cDNA synthesis followed protocols previously described for RNAseq analysis (Zhang et al., 2015c) and for quantitative real-time PCR (Zhou et al., 2010), respectively.

### RNAseq expression analysis of cellulose synthesis related genes

A previously published *de novo* assembled and validated RNAseq data set, derived from *trans*-differentiating cotyledon epidermal cells and underlying storage parenchyma cells harvested at 0, 3, and 12 h of cotyledon culture (Zhang et al., 2015c) was supplemented with an additional three replicates derived from epidermal peels. The cDNA sequence datasets of raw reads and assembled reference transcriptome library supporting the results are deposited at the European Nucleotide Archive (ENA) with the ENA accession number: PRJEB8906 (see http://www.ebi.ac.uk/ena/data/view/PRJEB8906). Transcripts were annotated using Blast2GO against NCBI Genbank (see Zhang et al., 2015c) and using Mapman Mercator (Lohse et al., 2014) against TAIR, Uniprot, TIGR, KOG, and Interpro scan.

Cellulose biosynthetic genes differentially expressed during wall labyrinth construction were identified using the following criteria: RPKM >1 for at least one of the specified sampling times; Log_2_ fold change in RPKM between one set of sampling times >1 with a false discovery rate (FDR) corrected *P* < 5% (determined using LimmaR; see Ritchie et al., 2015). Full-length CDS sequences of the identified differentially expressed genes are deposited in the Genbank database under the following accession numbers: *VfCesA1*, MG561952; *VfCesA3A*, MG561953; *VfCesA3B*, MG561954; *VfCesA6*, MG561955; *VfCSLD2*, MG561956; *VfCSLD3*, MG561957; *VfCSI1*, MG561958; *VfKOR1*, MG561959; *VfCOBRAL1*, MG561960; *VfCOBRAL2*, MG561961; *VfCOBRAL7*, MG561962.

### Quantitative real-time PCR of specified differentially expressed cellulose biosynthetic genes

Four housekeeping gene candidates were identified based on their RNAseq temporal expression profiles (Supplementary Figure S2). These were *elongation factor 2*-α (*VfEF2*α*), NADH dehydrogenase subunit 4* (*VfNADHD4*), *60S ribosomal protein subunit L2* (*Vf60SL2*), and *multi-domain cyclophilin type peptidyl-prolyl cis-trans isomerase G* (*VfPPlaseG*). The housekeeping genes were further validated using GeNorm analysis of real-time PCR data derived from cDNA of epidermal peels that collectively yielded a reliable reference *M* < 0.15 (Vandesompele et al., 2002).

Primers for the reference genes and specified differentially expressed cellulose biosynthetic genes (*VfCesA1, VfCesA3A, VfCesA3B, VfCesA6, VfCSL-D2*, and *VfCSL-D3*) were designed using Primer 3 plus (Whitehead Institute for Biomedical Research, USA) and synthesized by Sigma-Aldrich Australia (see Supplementary Table S3 for primer sequences). Real-time PCR was conducted on cDNA derived from epidermal peels to obtain temporal expression profiles of the specified differentially expressed cellulose biosynthetic genes normalized against the four house-keeping genes using the ΔΔCt method.

## Results

Upon transferring *V. faba* cotyledons to a MS culture medium, their adaxial epidermal cells spontaneously undergo *trans*-differentiation to a TC morphology characterized by developing a reticulate wall labyrinth polarized to, and deposited on, the cytoplasmic face of the original outer periclinal wall. This wall labyrinth is separated from the original wall by a narrow band of electron-dense material and is comprised of two structurally distinctive components; a uniform wall layer from which WI papillae arise (Figure 1B). Since cellulose is essential for WI papillae construction (Talbot et al., 2007), we proposed cellulose microfibril deposition could play an important role in underpinning construction of the uniform wall layer. To this end, cellulose microfibril organization within the uniform wall layer was visualized by FESEM and compared to that of the original primary wall and WI papillae.

### Cellulose microfibril organization differs substantially between original and uniform wall layers and wall ingrowth papillae

**Figure 3:**
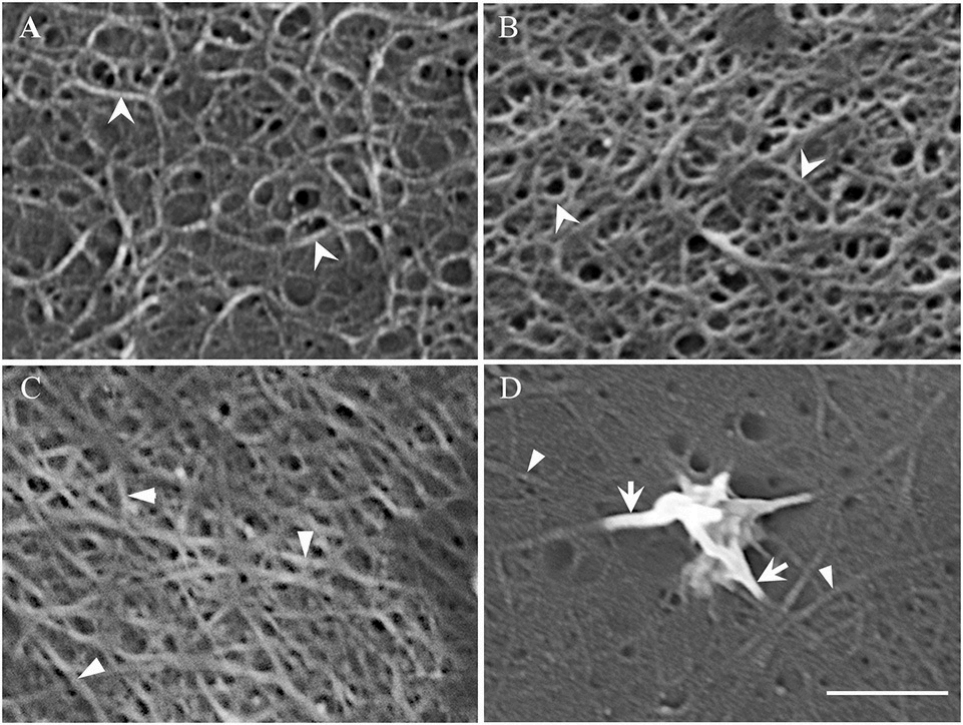
Field emission scanning electron micrographs of cellulose microfibrils viewed from the cytoplasmic face of the outer periclinal wall of adaxial epidermal cells. **(A,B)** Micrographs of the original wall layer obtained from **(A)** freshly harvested cotyledons and **(B)** cotyledons cultured for 15 h on 100 μM DPI to block uniform wall layer formation. **(C,D)** Micrographs of a fully-developed uniform wall layer obtained from cotyledons cultured for 15 h on MS medium alone. **(D)** In order to improve visualization of the thickened cellulose microfibrils proximal to the nascent WI papilla, excess electron charging of their surfaces was minimized by reducing the contrast setting from 43 to 30%. Cellulose microfibrils of the original **(A,B)** and uniform wall **(C,D)** layers are marked by arrowheads and darts, respectively. Arrows mark the thickened cellulose microfibrils that converge and intertwine to form WI papillae **(D)**. Bar, 150 nm **(A–C)** and 100 nm **(D)**.

Acid cleared outer periclinal walls of epidermal peels were examined by FESEM to image cellulose microfibrils prepared from cotyledons freshly harvested or cultured for 15 h on MS media in the presence/absence of diphenyleneiodonium (DPI) known to selectively block deposition of the uniform wall layer (Andriunas et al., 2012). At 15 h of cotyledon culture, uniform wall layer development was completed (Zhang et al., 2015c). Imaging cleared outer periclinal cell walls of epidermal peels sampled from freshly harvested cotyledons or from those cultured for 15 h on MS medium containing DPI, identified a loosely organized layer of curved cellulose microfibrils overlaying a relatively densely packed ring-like lattice of cellulose microfibrils in freshly harvested cotyledons (Figure 3A). This pattern of cellulose microfibril organization resembles that detected in primary walls of expanding mesophyll cells (Fujita and Wasteneys, 2013). However, only the densely packed ring-like lattice of cellulose microfibrils remained once epidermal cell expansion had ceased in cotyledons cultured for 15 h on DPI (Figure 3A vs. Figure 3B). This suggests that the loosely organized layer of curved cellulose microfibrils likely functions as a tension-bearing entity supporting anisotropic growth of the expanding epidermal cells (see Supplementary Table S4; Crowell et al., 2011).

In contrast, cellulose was organized into a less dense network of linear microfibrils in the fully-developed uniform wall layer (Figure 3C vs. Figure 3B) in epidermal cells of cotyledons cultured for 15 h on MS medium alone (Figures 3C,D). Immunocytochemical observations of cellulose distribution across the wall labyrinth would suggest that the observed cellulose microfibrils are spread throughout the width of the uniform wall layer (Vaughn et al., 2007). Diameters of cellulose microfibrils deposited in the original and uniform wall layers were identical (Table 1) and comparable to diameters of cellulose microfibrils in primary cell walls detected by atomic force microscopy (Ding et al., 2014; Zhang et al., 2016). In contrast, WI papillae arose from converging cellulose microfibrils, the diameters of which increased 3-fold proximal to nascent WI papillae (Figure 3D, Table 1).

**Table 1:** Diameters of cellulose microfibrils located in original and uniform wall layers, distal from, and proximal to, developing wall ingrowth papillae.

**Cellulose microfibril diameters (nm) located in wall components of epidermal cells following cotyledon culture as specified:**			
**Freshly harvested**	**Cultured on DPI**	**Cultured on MS medium alone**	
**Original wall layer**		**Uniform wall layer**	**Wall ingrowth papillae**
5.3 ± 0.5	4.2 ± 0.3	4.8 ± 0.3	13.2 ± 0.6^**^

*Cellulose microfibril diameters were measured using FESEM micrographs (see Figure 3) of the original wall layer obtained from freshly harvested cotyledons and cotyledons cultured for 15 h on 100 μM DPI to block uniform wall layer formation and of the uniform wall layer and developing wall ingrowth papillae obtained from cotyledons cultured for 15 h on MS medium alone. Means ± SEs of four replicate cotyledons with a minimum of 25 microfibrils measured per replicate (n = 100). Asterisk indicates a significant difference (Student's t-test*,

** *P < 0.01)*.

The distinctly different pattern, and relatively low density, of cellulose microfibrils in the uniform wall layer compared to those in the original primary wall (Figure 3C vs. Figure 3B) raised questions about regulation of their deposition and their role in assembling the uniform wall layer. Additionally, the pronounced increase in diameters of uniform wall layer cellulose microfibrils that form WI papillae points to a step change in cellulose biosynthesis machinery in transitioning from uniform wall layer to WI papillae assembly. Findings of experiments designed to address these questions as follows.

### Uniform wall layer deposition is microtubule independent

During *trans*-differentiation of cotyledon epidermal cells to a TC morphology, the aligned cortical microtubules, located in the outer periclinal region of precursor epidermal cells, are reorganized into a randomized array (Zhang et al., 2015b). Given that microtubules can influence cellulose microfibril deposition and hence cell wall morphology (McFarlane et al., 2014), it is conceivable that microtubules regulate the altered organization of cellulose microfibrils in transitioning from original wall to uniform wall layer deposition (see Figure 3). This proposition was tested by culturing cotyledons on MS media containing oryzalin or taxol that we have previously shown to respectively, depolymerize or stabilize the cortical microtubule array of the epidermal cells (Zhang et al., 2015b) and observing effects on uniform wall layer deposition and its cellulose microfibril organization.

**Figure 4:**
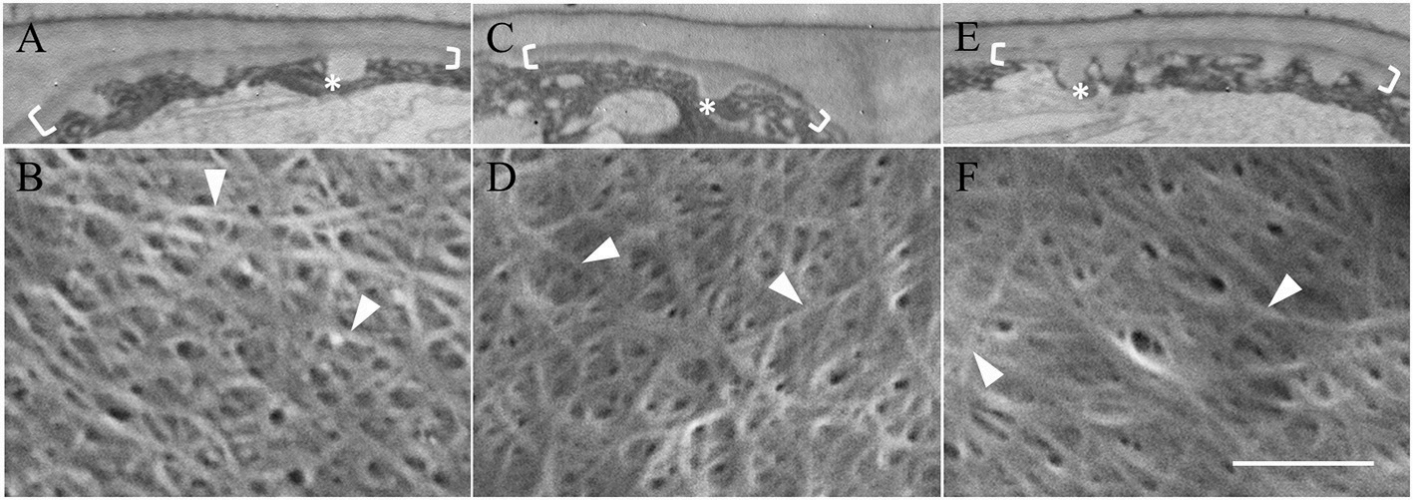
Effect of oryzalin and taxol on uniform wall layer structure and cellulose microfibril organization. Cotyledons were cultured in MS medium alone **(A,B)** or MS medium containing 20 μM oryzalin **(C,D)** or 5 μM taxol **(E,F)** for 15 h at 26°C following being held at 4°C for 4 h to ensure cellular uptake of the pharmacological agents before induction of ETC development on transfer to 26°C (see Zhang et al., 2015b). **(A,C,E)** Transmission electron micrographs of transverse sections of the outer periclinal walls of adaxial epidermal cells. Brackets mark the uniform wall layer and asterisks mark wall ingrowth (WI) papillae. **(B,D,F)** FESEM micrographs showing cellulose microfibrils deposited in the uniform wall layer (darts). Bar, 5 μm in **(A,C,E)** and 150 nm in **(B,D,F)**.

Neither depolymerizing (oryzalin) nor stabilizing (taxol) the cortical microtubule array (see Zhang et al., 2015b) exerted any detectable impact on uniform wall layer structure (Figure 4A cf. Figures 4C,E) or development deduced from cell percentages containing an uniform wall layer and its final thickness (Table 2). This outcome equally applied to the organization and diameter of cellulose microfibrils deposited in the uniform wall layer (Figure 4B cf. Figures 4D,F, Table 2). In contrast, oryzalin switched cell expansion from anisotropic to isotropic growth (Supplementary Table S4), indicating cellulose deposition in the expanding original wall was directed by their aligned cortical microtubule array (Zhang et al., 2015b).

**Table 2:** Effect of oryzalin or taxol on uniform wall layer deposition and cellulose microfibril diameters located in the uniform wall layer and developing wall ingrowth papillae.

**Treatment**	**Uniform wall layer:**		**Cellulose microfibril diameter (nm) in:**	
	**Cells (%)**	**Thickness (nm)**	**Uniform wall layer**	**Wall ingrowth papillae**
Control	90 ± 1	310 ± 15	4.5 ± 0.4	12.6 ± 0.8
Oryzalin	93 ± 4	299 ± 11	4.8 ± 0.5	13.9 ± 1.0
Taxol	92 ± 4	302 ± 13	4.6 ± 0.5	13.2 ± 1.2

*Cotyledons were cultured for 15 h at 26°C ± 20 μM oryzalin or ± 5 μM taxol following being held at 4°C for 4 h to ensure cellular uptake of the pharmacological agents prior to the rapid induction of TC development on transfer to 26°C (Zhang et al., 2015b). Data are means ± SEs with 60 cells scored from four replicate cotyledons to estimate percent cells containing a uniform wall layer (n = 4) and its thickness (n = 60); 100 measurements across four replicate cotyledons to measure cellulose microfibril diameters (n = 100)*.

### A cellulose scaffold is not essential for uniform wall layer deposition

The relatively low density of cellulose microfibrils in the uniform wall layer (Figure 3C vs. Figure 3B, Table 3) pointed to the possibility that, in contrast to WI papillae formation (Talbot et al., 2007), cellulose may not be essential for its construction. To test this proposition, cellulose biosynthesis was attenuated by culturing cotyledons on 2,6-dichlorobenzonitrile (DCB) that acts by immobilizing cellulose synthase complexes in the plasma membrane (DeBolt et al., 2007). As a positive control, to mitigate against any confounding effects of blocking WI papillae development by DCB (Talbot et al., 2007), cotyledons also were cultured on the Ca^2+^ chelator, BAPTA (1, 2-bis(o-aminophenoxy)ethane-N,N,N',N'-tetraacetic acid) to dampen the cytosolic Ca^2+^ signal regulating WI papillae formation (Zhang et al., 2015a).

**Table 3:** Relative densities and total amounts of cellulose microfibrils deposited in the original and uniform wall layers of adaxial epidermal cells of cultured *V. faba* cotyledons.

**Relative cellulose microfribril densities (arbitrary units) in:**		**Relative cellulose microfibril amounts (arbitrary units) in:**	
**Original wall layer**	**Uniform wall layer**	**Original wall layer**	**Uniform wall layer**
884 ± 12	88 ± 3^**^	12,995	1,804

*Relative cellulose densities were determined from FESEM micrographs of the outer periclinal wall of adaxial epidermal cells. Observations of the original wall were derived from freshly-harvested cotyledons and cotyledons cultured for 15 h on a MS medium containing 100 μM DPI to block formation of the uniform wall layer. Observations of the uniform wall layer were derived from cotyledons cultured for 15 h on MS medium alone. Means ± SEs of 8 replicate cotyledons with 50–75 measurements per cotyledon. Asterisk indicates a significant difference (Student's t-test*,

** *P < 0.01). Relative total amounts of cellulose microfibrils were estimated as the product of their relative densities and respective volumes of the original and uniform wall layers deposited during the culture period (Figure 8A)*.

**Figure 5:**
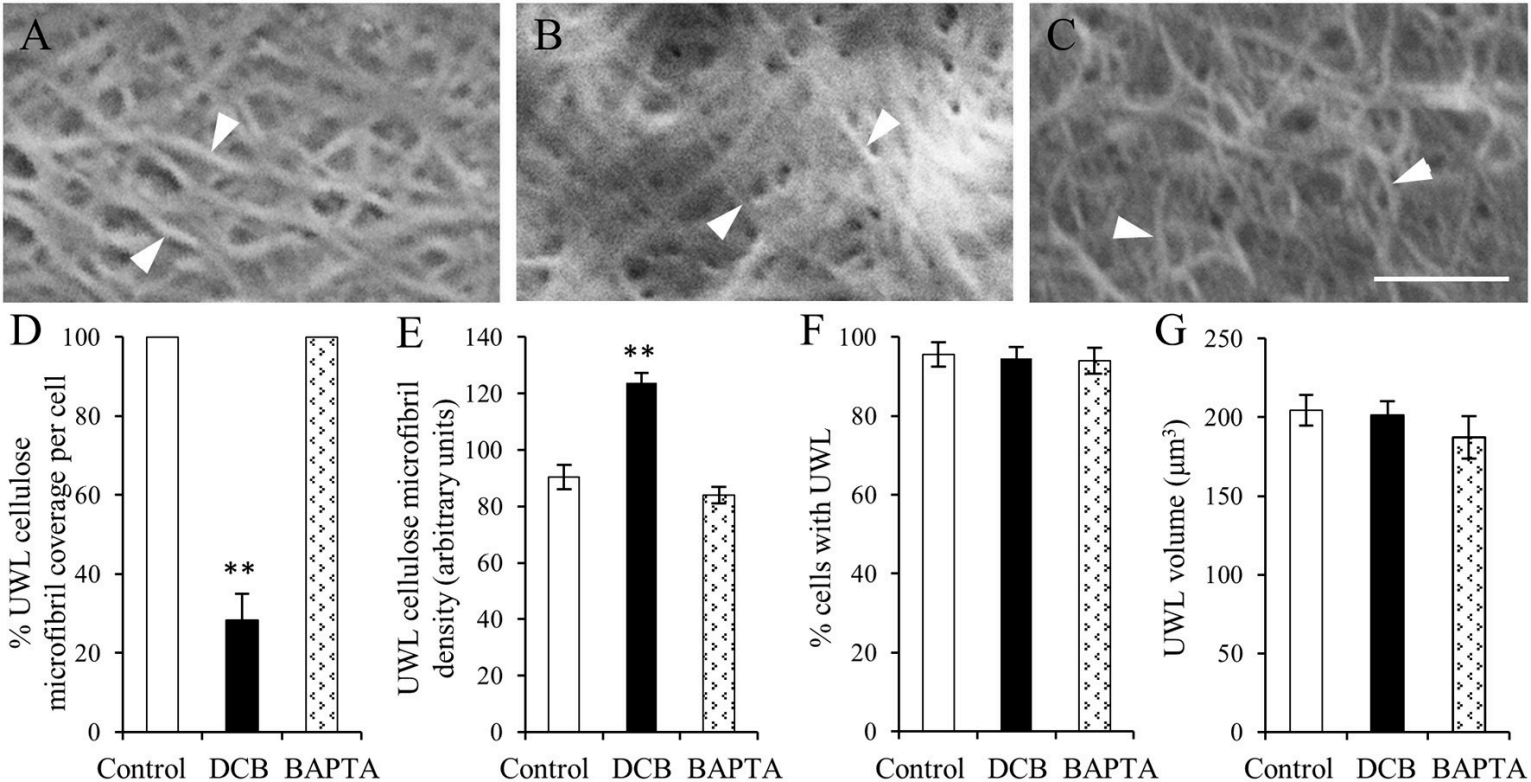
The effect of DCB or BAPTA on, cellulose deposition in, and formation of, the uniform wall layer **(A–C)**. FESEM micrographs of cellulose microfibrils (darts) viewed from the cytoplasmic face of the uniform wall layer in cotyledons cultured for 15 h on MS medium alone **(A)**, MS medium plus 5 μM DCB **(B)** or MS medium plus 600 μM BAPTA **(C)**. Note **(B)** is illustrating microfibrils within patches overlaying the original wall resulting from DCB treatment. Bar, 150 nm. **(D–G)** Effect of DCB and BAPTA on the uniform wall layer with respect to **(D)** percent coverage with cellulose microfibrils, **(E)** relative densities of cellulose microfibrils determined across the entire outer periclinal surface for control and BAPTA-treated cells and for the patches of cellulose microfibrils formed in response to DCB treatment (for more information, see Materials and Methods), **(F)** percent epidermal cells forming the uniform wall layer, and **(G)** its volume per cell. Means ± SEs (bars) of four to five replicate cotyledons, 40 to 200 determinations per replicate depending upon type of measurement. Asterisk indicates a significant difference (Student's *t*-test, ^**^P < 0.01) with percentage data arc sine transformed for the analysis.

The organization of cellulose microfibrils located within the uniform wall layer was comparable across all treatments (Figures 5A–C). However, DCB severely restrained (by 72%) cellulose coverage across the uniform wall layer (Figure 5D) to circular patches with diameters of 8.9 ± 0.2 μm (Supplementary Figure S3). Within these patches, densities of cellulose microfibrils were increased by 46% compared to control and BAPTA treatments (Figure 5E). This response combined with impacts on cellulose coverage (Figure 5D), but not on the volume (Figure 5G), of the uniform wall layer provided an estimate that DCB decreased cellulose microfibril deposition by 58%. These impacts on cellulose microfibril deposition are consistent with DCB immobilizing and aggregating cellulose synthase complexes into discrete patches on the plasma membrane (DeBolt et al., 2007). In contrast, exposure to BAPTA exerted no effect on uniform wall layer cellulose microfibril coverage, densities (Figures 5D,E) or volume (Figure 5G). Thus, despite differences in amounts of cellulose microfibrils deposited in the uniform wall layer of epidermal cells exposed to DCB and BAPTA, and their shared inhibitory action on WI papillae formation (Supplementary Figure S4 and for more information, see Talbot et al., 2007; Zhang et al., 2015a), neither DCB nor BAPTA prevented uniform wall layer deposition (Figures 5F,G). Collectively these data suggest that, in contrast to WI papillae (Talbot et al., 2007), development of the uniform wall layer does not depend upon deposition of a cellulose scaffold.

A sub-population of thickened cellulose microfibrils of the uniform wall layer converged to form WI papillae (Figure 3D, Table 1). This suggested that these microfibrils may be precursors of the cellulose scaffold known to be essential for WI papillae development as shown by blocking their formation with cellulose biosynthesis inhibitors (Supplementary Figure S4; Talbot et al., 2007).

### Cellulose deposition in the uniform wall layer is essential for wall ingrowth papillae formation

An experimental design to test the hypothesis that the uniform wall layer, and specifically the deposited cellulose microfibrils, are essential for WI construction rested on the knowledge that epidermal cells retain their capacity to form WI papillae following a 15-h blockade with reversible pharmacological agents (e.g., BAPTA—Zhang et al., 2015a) and that DCB is a reversible inhibitor of cellulose biosynthesis (Montezinos and Delmer, 1980). To this end, cotyledons were cultured on BAPTA or DCB for 15 h. Thereafter, DCB and BAPTA residues were removed from the cotyledons by a washing regime before transferring the cotyledons to a fresh MS medium for a further 15 h. Together with a control comprised of cotyledons cultured for 15 h on MS medium alone, these treatments were used to explore the relationship between WI papillae formation and cellulose microfibril presence in the uniform wall layer.

**Figure 6:**
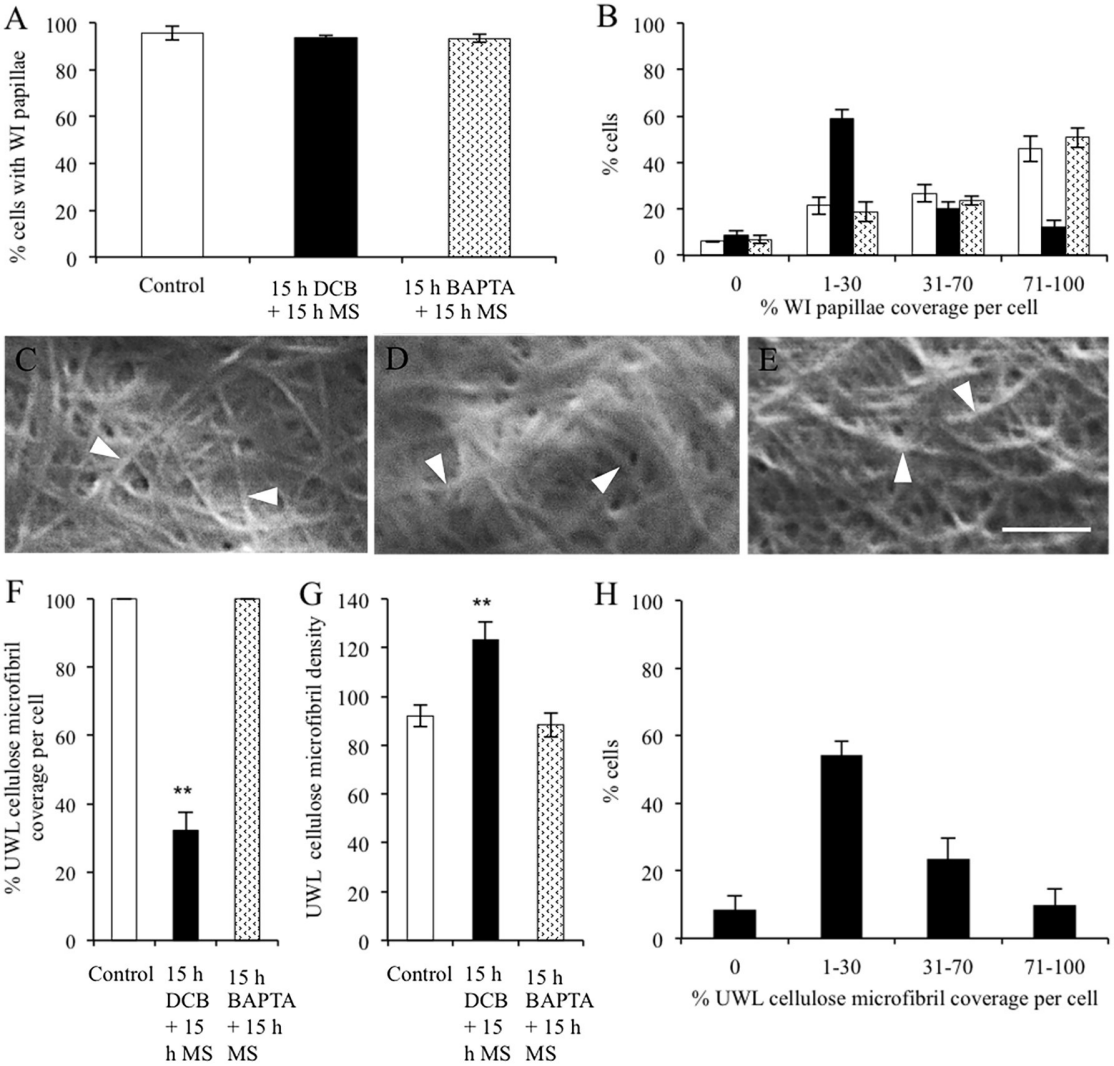
Effect of recovery from DCB or BAPTA treatments on the relationship between wall ingrowth papillae formation and cellulose microfibril deposition. Cotyledons were cultured on MS medium ± 5 μM DCB or 600 μM BAPTA for 15 h. Thereafter, following 3 × 10-min washes in MS medium, the cotyledons were transferred to fresh MS medium alone for a further 15 h. **(A,B)** Recovery from treatment on wall ingrowth (WI) papillae formation as determined by percent epidermal cells **(A)** containing WI papillae or **(B)** within specified relative ranges of WI papillae coverage across the outer periclinal cell wall surface. Means ± SEs (bars) of four replicate cotyledons with a 100 cells scored per replicate (*n* = 4). Asterisk indicates a significant difference (Student's *t*-test, ^**^P < 0.01) with percentage data arc sine transformed for the analysis. **(C–E)** FESEM micrographs of cellulose microfibrils (darts) deposited in the uniform wall layer following recovery from **(C)** MS medium alone, **(D)** DCB, or **(E)** BAPTA treatments. Bar, 150 nm. **(F–H)** Cellulose microfibril organization in the uniform wall layer following treatment recovery in terms of: **(F)** cellulose microfibril coverage per cell **(G)** relative densities of cellulose microfibrils **(H)** percent of the DCB treated cells exhibiting specified ranges of relative cellulose coverage across their outer periclinal cell wall surface. Means ± SEs (bars) of four replicate cotyledons with 10–150 determinations per replicate depending on the type of measurement. Asterisk indicates a significant difference (Student's *t*-test, ^**^P < 0.01) with percentage data arc sine transformed for the analysis.

The percentage of epidermal cells forming WI papillae was fully restored in the DCB and BAPTA recovery treatments (Figure 6A). However, marked differences between treatments were apparent in the percent coverage of the adaxial cell surface with WI papillae. The control and BAPTA treatments exhibited similar profiles with 67% of cells exhibiting a coverage of WI papillae >30%. In contrast, for the DCB recovery treatment, this level of WI papillae coverage was reduced to 29% of cells (Figure 6B). Uniform wall layer cellulose microfibrils exhibited comparable coverages and densities to those found after 15 h within each pharmacological treatment (Figures 6C–G vs. Figures 5A–E). This finding suggests that no further cellulose was deposited into the uniform wall layer during the subsequent 15-h recovery period. Of most significance, for the DCB treatment, the relative coverage profiles of WI papillae closely aligned with that for cellulose microfibrils in the uniform wall layer (Figure 6H cf. Figure 6B; *r*^2^ = 0.98); a relationship consistent with WI papillae construction being dependent on precursor cellulose microfibrils in the uniform wall layer laid down as patches.

Potential contributions of enzymes encoded by cellulose biosynthetic-related genes to the observed changes in cellulose microfibril organization and diameters during formation of the uniform wall layer and WI papillae were examined by interrogating a validated RNAseq data set (Zhang et al., 2015c) for correlative changes in their transcript abundance.

### Expression of cellulose biosynthetic-related genes during construction of the original and uniform wall layers and wall ingrowth papillae

**Figure 7:**
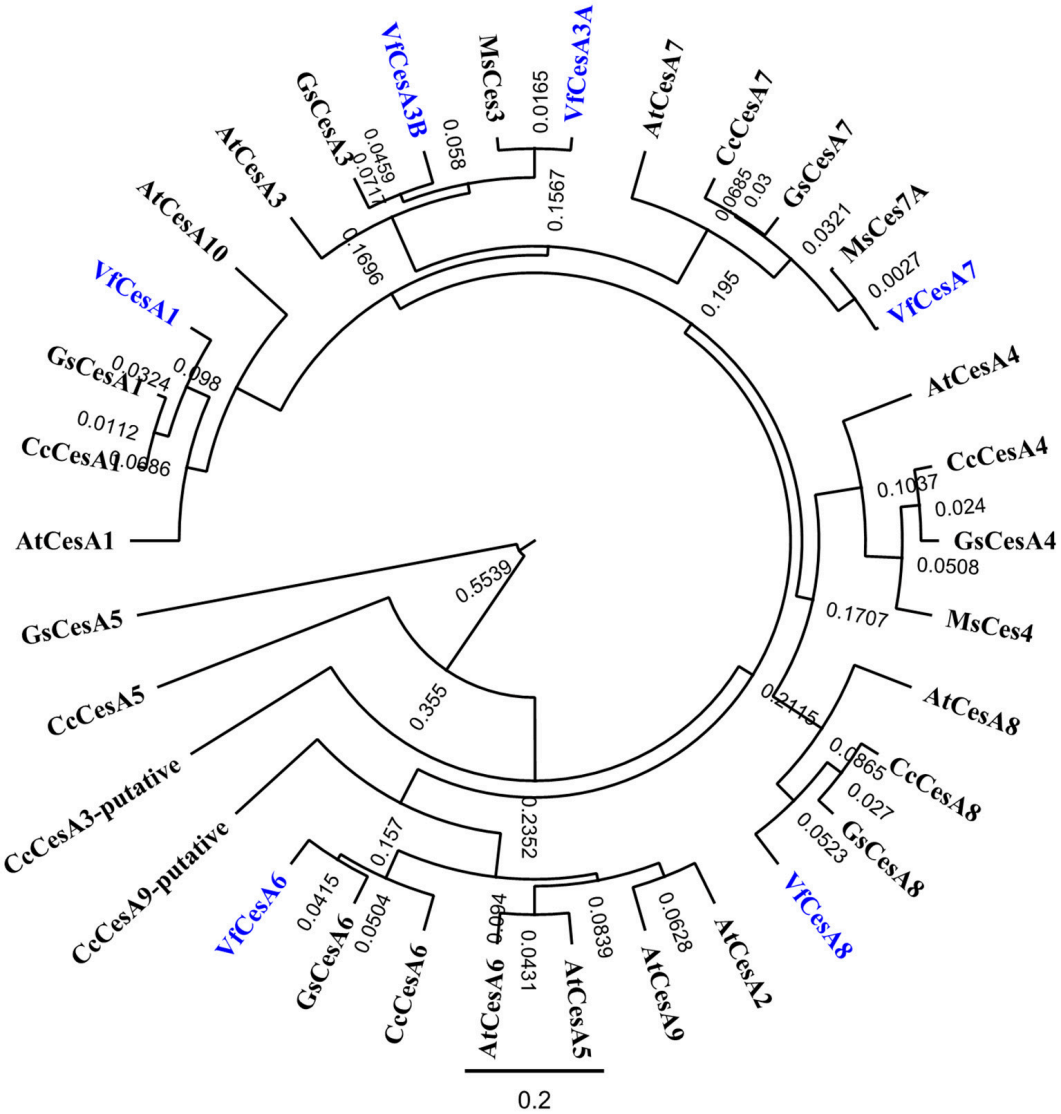
Phylogenetic analyses of *V. faba* cellulose synthesis genes in relation to their homologs from various species. Full length amino acid sequences of VfCesA1 (GenBank accession number MG561952), VfCesA3A (MG561953), VfCesA3B (MG561954) and VfCesA6 (MG561955), and partial amino acid sequences of VfCesA7, VfCesA8 were aligned to cellulose synthase sequences from *Cajanus cajan* (Cc), *Arabidopsis thaliana* (At), *Glycine soja* (Gs), and *Medicago sativa* (Ms). The tree was constructed by Geneious 8 (Biomatters Ltd.) and sequences were aligned using ClustalOmega. The length of the lines connecting the sequences is proportional to the estimated amino acid substitutions/site between these sequences. Bootstrp values from 1,000 iterations are shown. All sequences were obtained from NCBI (http://www.ncbi.nlm.nih.gov). Accession numbers include CcCesA1 (KYP70177.1), CcCesA3-putative (KYP59440.1), CcCesA4 (KYP43719.1), CcCesA5 (partial, KYP74142.1), CcCesA6 (KYP69388.1), CcCesA7 (KYP51458.1), CcCesA8 (KYP38913.1), CcCesA9-putative (partial KYP50958.1), AtCesA1 (NP_194967.1), AtCesA2 (NP_195645.1), AtCesA3 (NP_196136.1), AtCesA4 (NP_199216.2), AtCesA5 (NP_196549.1), AtCesA6 (NP_201279.1), AtCesA7 (NP_197244.1), AtCesA8 (NP_567564.1), AtCesA9 (NP_179768.1), AtCesA10 (NP_180124.1), GsCesA1 (KHN08123.1), GsCesA3 (KHN37622.1), GsCesA4 (KHN47729.1), GsCesA5 (partial, KHN12485.1), GsCesA6 (KHN34038.1), GsCesA7 (KHN42479.1), GsCesA8 (KHN48007.1), GsCesA9-putative (partial, KHN42500.1), MsCesA3 (AII73575.1), MsCesA4 (AII73573.1), and MsCesA7A (AII73574.1).

Homologues of full-length amino acid sequences for the primary cell wall cellulose synthases, VfCesA1, VfCesA3A and VfCesA6 were detected along with partial sequences of an additional distinctive VfCesA3B and secondary wall cellulose synthase CesAs, VfCesA7, and VfCesA8 (Figure 7; McFarlane et al., 2014). In contrast to the primary cell wall CesAs, expression levels of *VfCesA7* and *VfCesA8* were < 1 RPKM throughout wall labyrinth development (see Supplementary Table S5) and hence were excluded from further analysis (see Materials and Methods). The partial sequence of the additional distinctive CesA3 transcript was used as a backbone to clone, using a combination of PCR and 3′ RACE, a full-length amino acid sequence annotated as VfCesA3B (Supplementary Figure S5). Differences in nucleotide bases were distributed randomly throughout *VfCesA3A* and *VfCesA3B* sequences (Supplementary Figure S6). This suggests that these transcripts were transcribed from distinct genes rather than being the product of alternative splicing in which particular exons may be included or excluded from the final processed transcript arising from the same gene (Leff et al., 1986). Also detected were transcripts of full-length sequences encoding *V. faba* homologs of cellulose synthase-like enzymes, *CSL-D2* and *CSL-D3* (Supplementary Figure S7), CesA associated proteins, *cellulose synthase interactive protein 1(CSI1)* and *KORRIGAN* (*KOR*) and two GPI-anchored proteins, *COBRAL-1* and *COBRAL-7*, that facilitate cellulose biosynthesis (Supplementary Table S5; Schneider et al., 2016).

**Figure 8:**
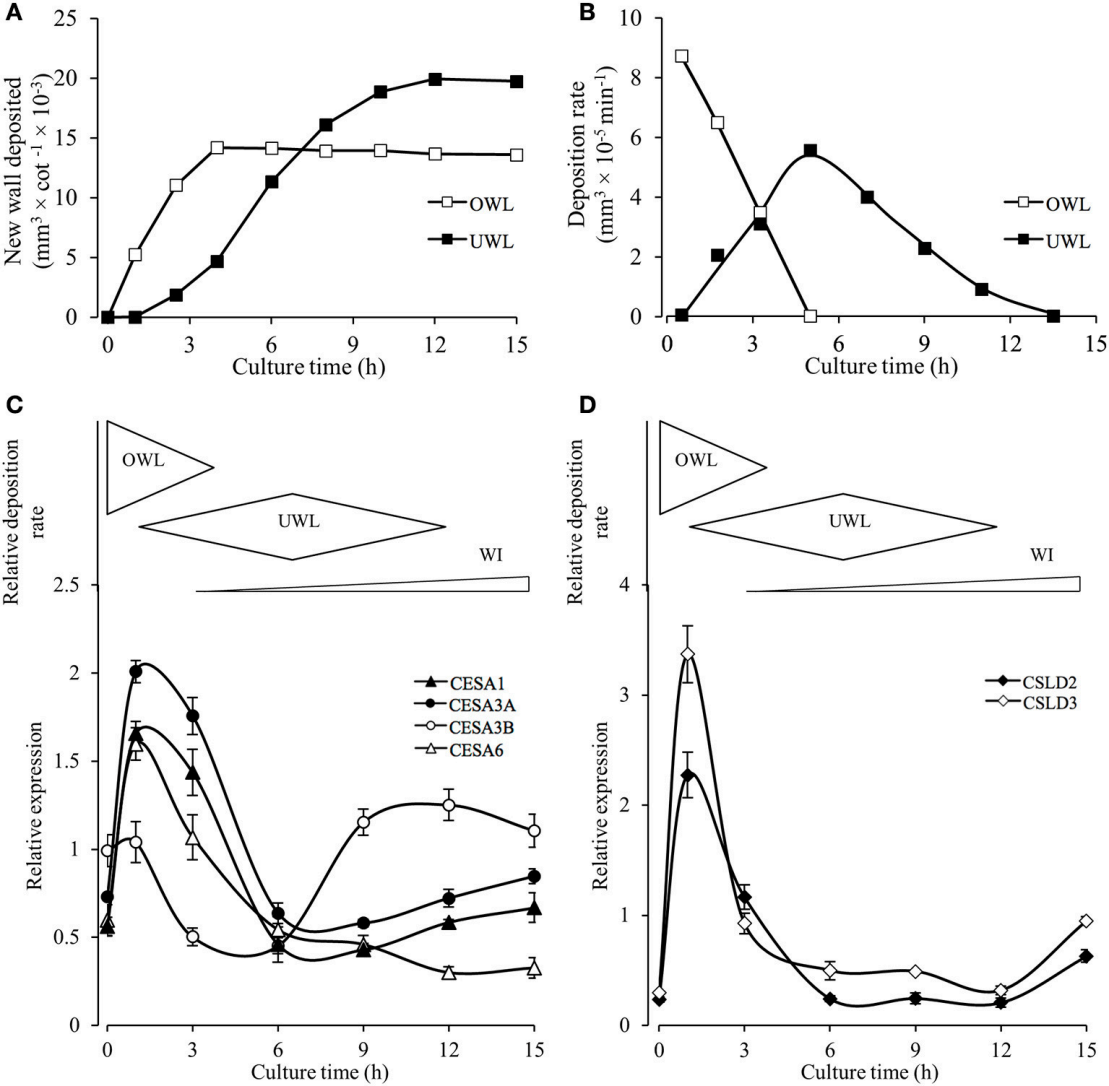
Temporal patterns of original and uniform wall layer deposition and expression profiles of cellulose biosynthesis related genes in the adaxial epidermal cells. **(A)** Temporal changes in newly deposited volumes of original (OWL) and uniform wall (UWL) layers per cotyledon (for wall volume derivations, see Materials and Methods and Supplementary Table S2). **(B)** Temporal changes in deposition rates of the original and uniform wall layers computed from wall volume estimates presented in **(A)**. **(C,D)** Cartoon above each panel shows relative deposition rates of original (OWL) and uniform (UWL) wall layers and wall ingrowth (WI) papillae per cultured cotyledon. Normalized real-time PCR estimates of transcript levels (for details, see Materials and Methods) in epidermal peels of **(C)** VfCesAs and **(D)** VfCSLs of cultured cotyledons. Means ± SEs (bars) of four replicate batches of adaxial epidermal peels (*n* = 4).

To further investigate a potential correlative relationship between expression of the identified *VfCesA* and *VfCSL* transcripts and deposition of wall material forming the original and uniform wall layers we established (i) a temporal profile of the volume and rate of deposition of new wall material across the 15-h culture period (Figures 8A,B) and (ii) a more detailed temporal expression profile of the identified *VfCesA* and *VfCSL* transcripts (Figures 8C,D). During the initial 2.5–4 h of cotyledon culture, the adaxial epidermal cells continued to expand, at a decreasing rate, without any detectable change in the cross-sectional area of their original wall (Supplementary Table S2). Such a relationship points to an ongoing deposition into the original primary wall (cf. Kutschera, 2008). Indeed, the volume of this newly deposited original wall material per cotyledon continued to accrue at a diminishing rate until 4 h of cotyledon culture (Figures 8A,B). Thus, on a cotyledonary basis, the early (initial 3–4 h) phases of induction and deposition of the uniform wall layer overlapped with ongoing original wall construction (Figures 8A,B). Peak construction rates of the two wall layers were displaced by 4 h with uniform wall layer deposition reaching completion by 12 h (Figures 8A,B).

The temporal expression profiles of primary wall *VfCesA* and *VfCSL* homologs showed that their expression peaked at 1 h of cotyledon culture corresponding with maximal rates of original wall deposition (Figures 8C,D cf. Figure 8B). Thereafter, transcript levels declined to be commensurate with those detected in freshly-harvested cotyledons by 6 h of culture with the initial decline being more precipitous for the *VfCSL* transcripts (Figure 8D cf. Figure 8C). The latter most closely followed the decreasing rates of original wall formation (Figure 8B). The slower decline in *CesA* transcript levels corresponds with that of the combined rates of constructing the original and uniform wall layers that halves by 6 h compared to 1 h of culture (Figure 8B). Post 6 h of culture there is a marked departure in these shared temporal expression patterns. Most notably *VfCesA3B* transcript levels more than doubled between 6 and 9 h while expression levels of *VfCesA1* and *VfCesA3*A show a slight upward trend between 9 and 15 h of cotyledon culture and *VfCesA6* expression exhibits a gradual decline (Figures 8C,D). The *VfCesA3B* temporal expression pattern maps with an increasing investment into WI papillae construction (Figure 8C).

## Discussion

### Overall interrelationship between formation of the original primary wall and the uniform wall layer

The uniform wall layer is deposited inward of a pre-existing outer periclinal primary wall (i.e., the original wall; Andriunas et al., 2012). Expression of *VfCesA1, VfCesA3A, B*, and *VfCesA6* throughout expansion of the original wall and wall labyrinth deposition is consistent with the wall labyrinth being of a primary origin (Vaughn et al., 2007). Moreover, identical diameters of cellulose microfibrils in the original and uniform wall layers (Table 1) correlated with unaltered ratios of transcript levels of these cellulose biosynthetic enzymes across formation of the original and uniform wall layers (Figures 8C,D). In contrast, cellulose microfibril deposition differed substantially between these wall layers characterized by a 7.2-fold reduction in the relative amounts of cellulose deposited in the uniform wall layer as relatively linear cellulose microfibrils (Figure 3C, Table 3). Amongst genes expressing ß 1–4 glucan synthases in the epidermal cells, this decrease in cellulose deposition was matched most closely by corresponding reductions in transcript levels of *VfCSL-D2* (6.7x) and *VfCSL-D3* (9x; see Figure 8D). Significantly, an analysis of Arabidopsis mutants of *csl-d2* and *csl-d3*, demonstrated that these ß 1–4 glucan synthases play a key role in the polarized deposition of cellulose essential for tip growth of root hairs (e.g., Galway et al., 2011). Unknown is whether they function as cellulose synthases or regulators of cellulose biosynthesis.

### Microtubule-independent deposited cellulose microfibrils do not form an essential scaffold for uniform wall layer construction

Although uniform wall layer construction exhibited characteristics of a primary wall (see above), pharmacological disruption of the re-modeled cortical microtubule network in the *trans*-differentiating epidermal cells (Zhang et al., 2015b) suggested it exerted no influence over the dramatically altered pattern of cellulose microfibril deposition in the uniform wall layer (cf. Schneider et al., 2016). This contrasts with deposition of the original wall where anisotropic expansion of the epidermal cells, and presumably the pattern of cellulose microfibril deposition (Baskin, 2005), was found to be dependent upon an aligned microtubule array; a finding consistent with the strong expression of *VfCSI1* encoding a protein that links cellulose synthase complexes to microtubules (Schneider et al., 2016). Since *VfCSI1* was strongly expressed throughout wall labyrinth construction, this suggests that its encoded protein likely was post-translationally decoupled from the cellulose synthase complexes once uniform wall layer deposition commenced. Microtubule independent deposition of uniform wall layer cellulose microfibrils is consistent with that for the subsequent formation of WI papillae (Zhang et al., 2015b) and secondary wall formation in differentiating tracheary elements (Li et al., 2016).

In the presence of the cellulose biosynthetic inhibitor, DCB (DeBolt et al., 2007), deposition of cellulose microfibrils in the uniform wall layer was restricted to patches (Supplementary Figure S3). In sharp contrast to blocking WI papillae construction (Talbot et al., 2007), the 58% reduction in cellulose microfibril deposition had no impact on uniform wall development. This suggests that the structural scaffold for uniform wall layer construction is contributed by matrix polysaccharides that could include pectins and xylogucans (Vaughn et al., 2007; Zhang et al., 2015c). For example, in cellulose-depleted walls of DCB-habituated cells, pectins (Sabba et al., 1999) and hemicelluloses (De Castro et al., 2015) have been found to serve as compensatory structural scaffolds. The significant role played by the matrix polysaccharide in assembling the uniform wall layer is consistent with an absence of any control being exerted by the cortical microtubule array.

### A sub-population of thickened uniform wall cellulose microfibrils is essential for wall ingrowth papillae construction

The ubiquitous relationship between a uniform wall layer from which WI papillae arise suggests a dependence of the latter on the former. The strong relationship between the cell coverage of DCB-induced patches of cellulose microfibrils and that of WI papillae (Figures 6H cf. Figure 6B) provided persuasive circumstantial evidence that the sub-population of uniform wall layer thickened cellulose microfibrils observed to converge (Figure 3D) represent precursors to form the inner cellulose scaffold essential for WI papillae construction (Talbot et al., 2007; Vaughn et al., 2007). A distinctive feature of this sub-population of uniform wall layer cellulose fibrils is a 3-fold increase in their diameters proximal to developing WI papillae. The enhanced microfibril thickness could confer mechanical strength to support vertical extension of WIs into the epidermal cell cytosol.

No evidence could be found for uniform wall layer cellulose microfibrils merging to form the thickened microfibrils (Figures 3C,D cf. Ding et al., 2014). Rather the gradual increase in their diameters to reach maxima on converging to form WI papillae (Figure 3D), suggests that the thickened microfibrils were generated by microfibrils aggregating coincident with coalescence of their glucan chains (i.e., a twinning mechanism—Oehme et al., 2015). This scenario depends upon: (i) cellulose synthase complexes being arranged in closely-packed clusters of three in the plasma membrane with each complex extruding cellulose microfibrils 4 nm in diameter (Ding et al., 2014; Oehme et al., 2015; Li et al., 2016); (ii) subtle changes in the surface properties of interacting microfibrils from each cellulose synthase complex in a cluster to favor thickening of the microfibrils, 13 nm in diameter (Oehme et al., 2015); (iii) accounting for the co-existence of two populations of cellulose fibrils deposited in the uniform wall layer.

A precedent for Requirement (i) is the recent finding of an increased cellulose microfibril aggregation between primary and secondary walls of *trans*-differentiating tracheary elements being linked with an increased density of cellulose synthase complexes (Li et al., 2016). In this context, we hypothesize that a high density of cellulose synthase complexes are constrained to tips of developing WI papillae as suggested by their spirally organized cellulose microfibrils being arranged perpendicularly to the underlying uniform wall layer (Talbot et al., 2007). To test this hypothesis, numbers of thickened cellulose microfibrils in a WI papillae can be estimated as follows. If the plasma membrane area covering the tip of the cellulose core of each WI papillae (i.e., 31,430 nm^2^ and see Talbot et al., 2007) was fully occupied by cellulose synthase complexes, 30 nm in diameter (Somerville, 2006), and these were organized in clusters of three to generate thickened cellulose microfibrils 13 nm in diameter (Ding et al., 2014), each WI papillae would contain an estimated 15 thickened cellulose microfibrils. This number approximately corresponds with cellulose microfibril numbers observed in WI papillae (e.g., see Figure 3D; Talbot et al., 2007).

Requirements (ii) and (iii) could be met by a temporally graduated change in the CesA composition of cellulose synthase complexes in transitioning from cellulose synthesis for uniform wall layer to WI papillae construction. This transition was characterized by a marked temporal shift in expression levels of genes encoding cellulose biosynthetic enzymes (Figures 8C,D). At 3 h of culture, cell wall biosynthesis is dominated by uniform wall layer construction. Here the ratios of transcript levels of *VfCesA1, VfCesA3A* + *VfCesA3B, VfCesA6* approximate the 1:1:1 ratio predicted for a cellulose synthase complex (Gonneau et al., 2014) with equal amounts of *VfCesA3A* and *VfCesA3B* transcript. Across 6 to 9 h a steep increase in *VfCesA3B* transcript levels occurred (Figure 8C) so that by 12 h, where cell wall synthesis is exclusively committed to WI papillae construction, there is a marked shift in *VfCesA* transcript ratios. Here *VfCesA3A* + *VfCesA3B*, transcript levels are 3 and 4 times greater than those of *VfCesA6* and *VfCesA1*, respectively with *VfCesA3B* dominating both within and between isoforms. Amino acid differences between the two CesA3 alleles are most pronounced in the zinc finger domain in which two highly conserved amino acids at D25 and N60 are substituted for E and K, respectively in VfCesA3B (Supplementary Figure S6). These amino acid substitutions could affect the hetero- and homodimerization of VfCesA3B with VfCesA1/VfCesA6 and with VfCesA3A, respectively that in turn could affect properties of the synthesized cellulose (McFarlane et al., 2014; Pysh, 2015). Therefore, the densely organized cellulose synthase complexes at the tips of WI papillae, in combination with the dominance and dimerization characteristics of VfCesA3B in the cellulose synthase complexes, collectively could contribute to forming thickened cellulose microfibrils essential for constructing WI papillae.

## Author contributions

JP and CO conceived and designed the research project. XX and H-MZ performed the experiments and compiled the data sets and assisted by JP and CO the interpretation of images and analysis of data. XX wrote the first draft of the manuscript that was revised by JP and reviewed by CO, XX, and H-MZ.

### Conflict of interest statement

The authors declare that the research was conducted in the absence of any commercial or financial relationships that could be construed as a potential conflict of interest.

## Abbreviations

CesA: cellulose synthase
BAPTA: 1, 2-bis(o-aminophenoxy)ethane-N,N,N',N'-tetraacetic acid
DCB: 6-dichlorobenzonitrile
DPI: diphenyleneiodium
FESEM: field emission scanning electron microscopy
MS: Murashige and Skoog
ROS: reactive oxygen species
RNAseq: RNA sequencing
SEM: scanning electron microscopy
SE: standard error of the mean
TC: transfer cell
TEM: transmission electron microscopy
WI: wall ingrowth papillae.
