# Supplementary material for: A Structurally Specialized Uniform Wall Layer is Essential for Constructing Wall Ingrowth Papillae in Transfer Cells
Source: Front Plant Sci. 2017 Dec 5;8:2035. doi: 10.3389/fpls.2017.02035 (PMC5723425; doi:10.3389/fpls.2017.02035)
Supplement: Supplementary file 1 [file DataSheet1.pdf]

Supplementary Materials

A Structurally Specialized Uniform Wall Layer is Essential for Constructing Wall Ingrowth Papillae in Transfer Cells

Xue Xia, Hui-Ming Zhang, Christina E Offler, John W Patrick*

***Correspondence:** John W Patrick: john.patrick@newcastle.edu.au

2. Supplementary Figures and Tables

2.1 Supplementary Figures

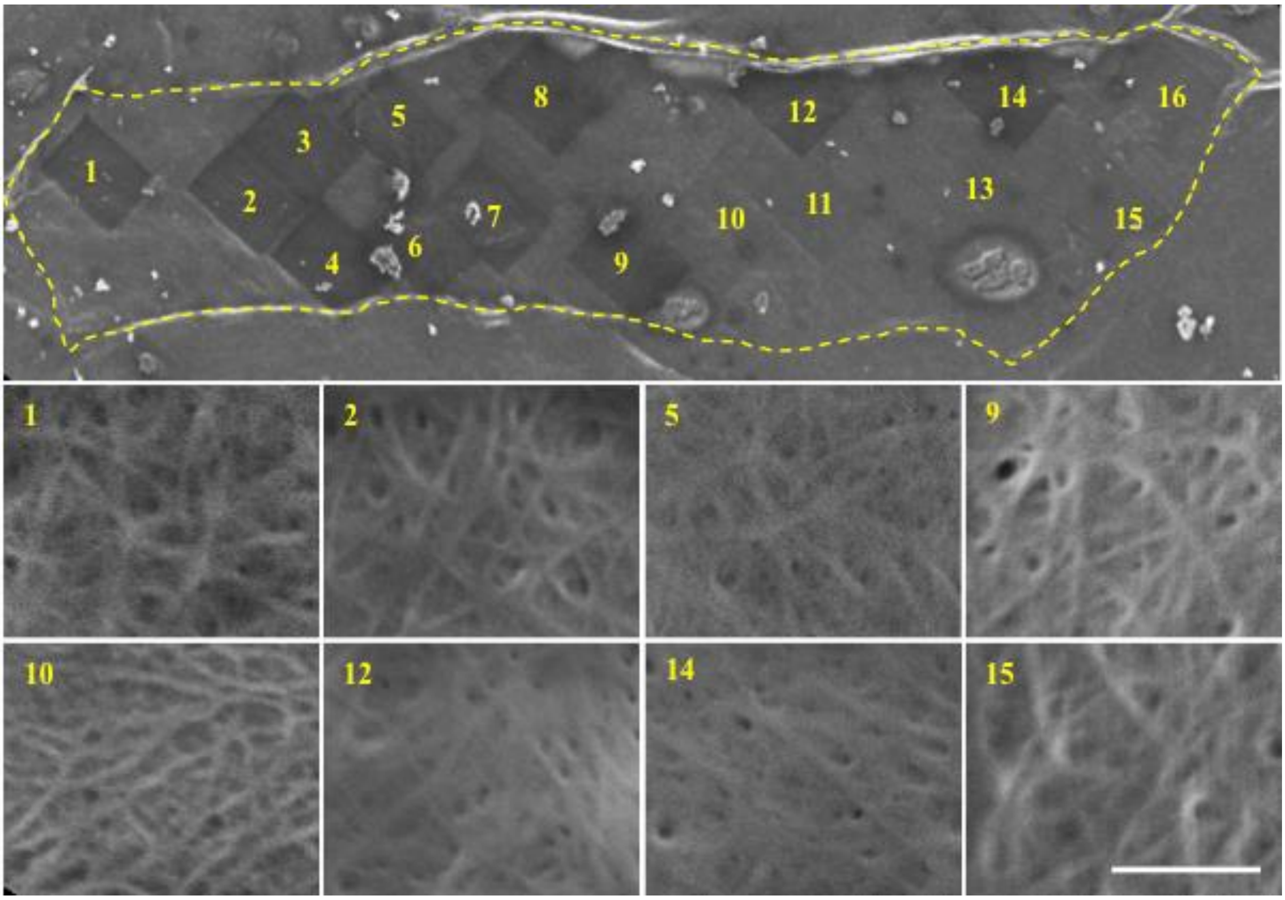

Supplementary Figure S1. Illustration of the sampling procedure to determine cellulose microfibril coverage per cell and densities in the uniform wall layer of *trans*-differentiating epidermal cells of cotyledons cultured for 15 h. The uppermost panel is a field emission scanning electron micrograph of a cleared cell (outlined by the dashed yellow line) showing randomly selected areas (labelled 1-16, sized 3.1 x 2.1 μm^2) that have been imaged at a higher power to visualize the cellulose microfibrils in the uniform wall layer, Examples of these higher magnification images are presented below with their numbering corresponding to the numbered patches in the micrograph of the cleared cell. Bar, 3 μm in whole cell micrograph and 80 nm in the higher power micrographs.

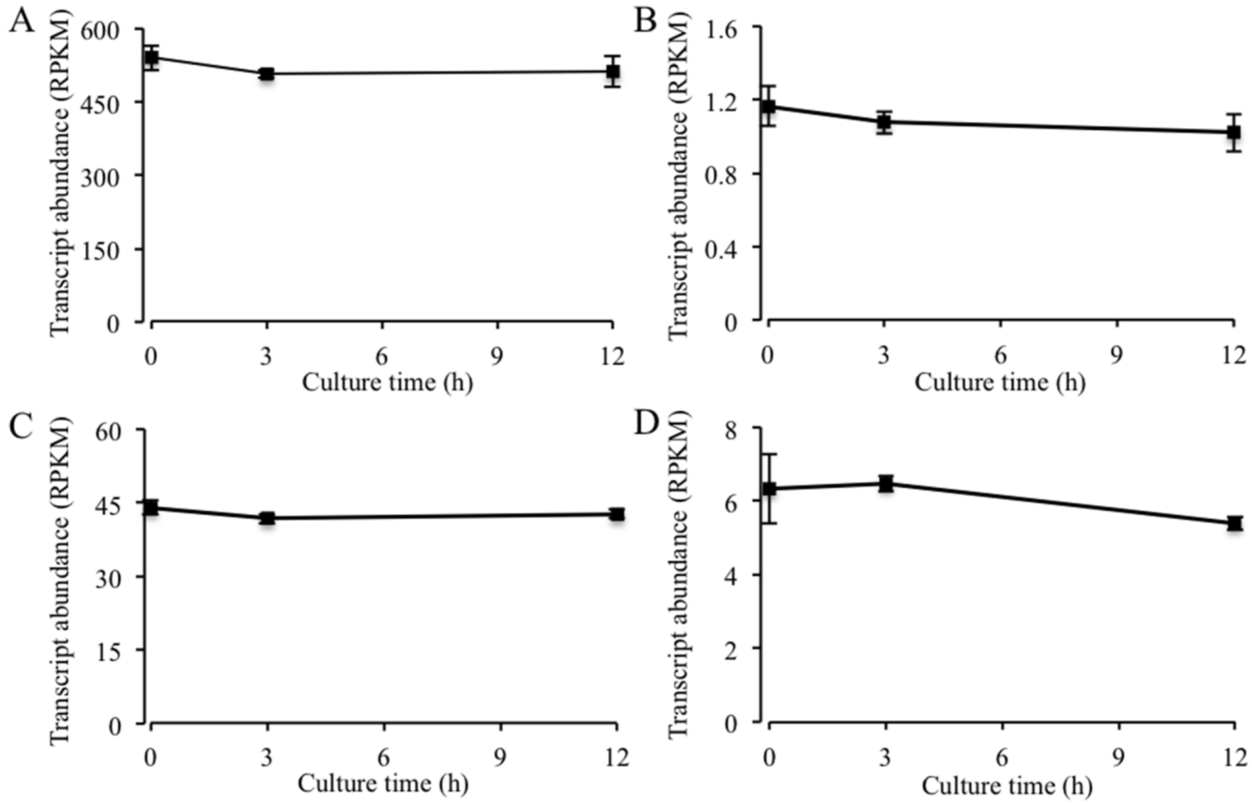

Supplementary Figure S2. Temporal expression profiles of housekeeping genes for normalization of real-time PCR studies of gene expression in adaxial epidermal cells of *V. faba* cotyledons cultured for specified times. (A) *Elongation factor 2-alpha (VfEF2α)*; (B) *NADH dehydrogenase subunit 4 (VfNADHD4)*; (C) *60S ribosomal protein subunit L2 (Vf60SL2)*; (D) *multi-domain cyclophilin type peptidyl-prolyl cis-trans isomerase G (VfPPlaseG)*. All candidates were further validated using real-time PCR to test their reliability as housekeeping genes (see Material and Methods for more details). Data are Means ± SEs of six replicate biological samples of adaxial epidermal cells (n = 6).

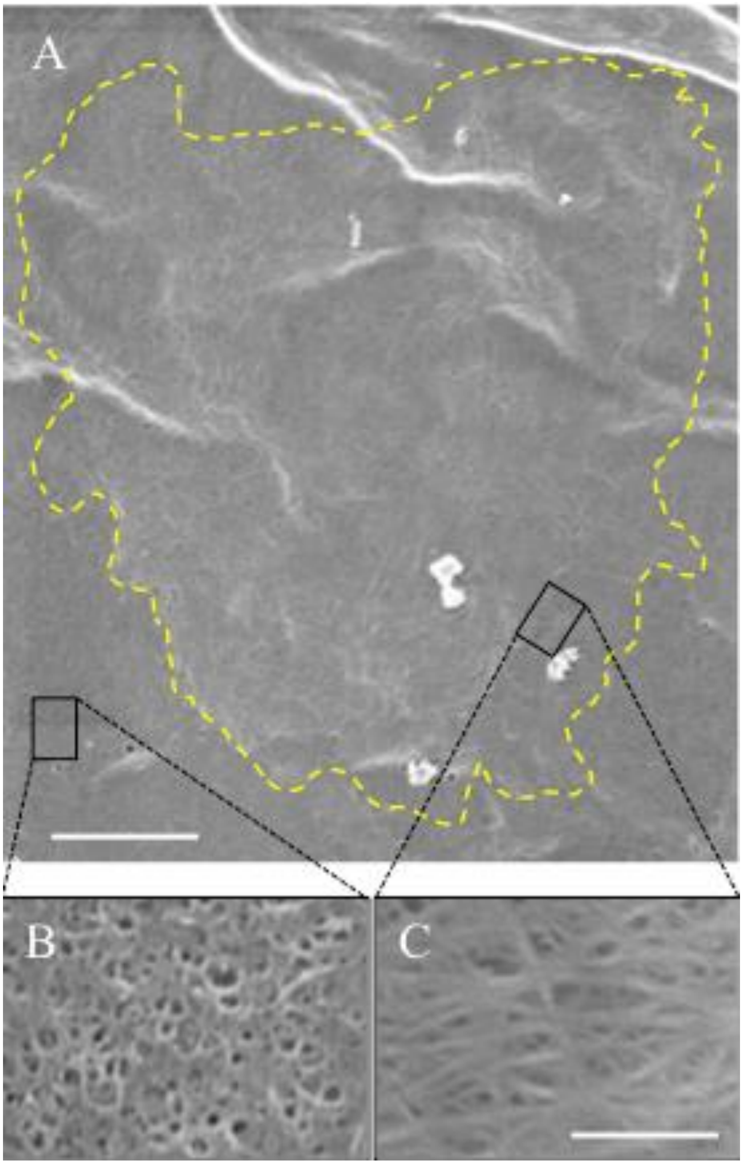

Supplementary Figure S3. Field emission scanning electron micrographs illustrating cellulose microfibrils deposited in the uniform wall layer of adaxial epidermal cells of *V. faba* cotyledons cultured for 15 h on 5 μM DCB. (A) Micrograph showing a patch of uniform wall layer cellulose microfibrils outlined by a dashed yellow line. (B, C) Higher power micrographs imaging cellulose microfibrils (B) outside the patch displaying an original wall configuration and (C) within the patch displaying a uniform wall layer configuration, Bar, 2 μm in A and 400 nm in B and C.

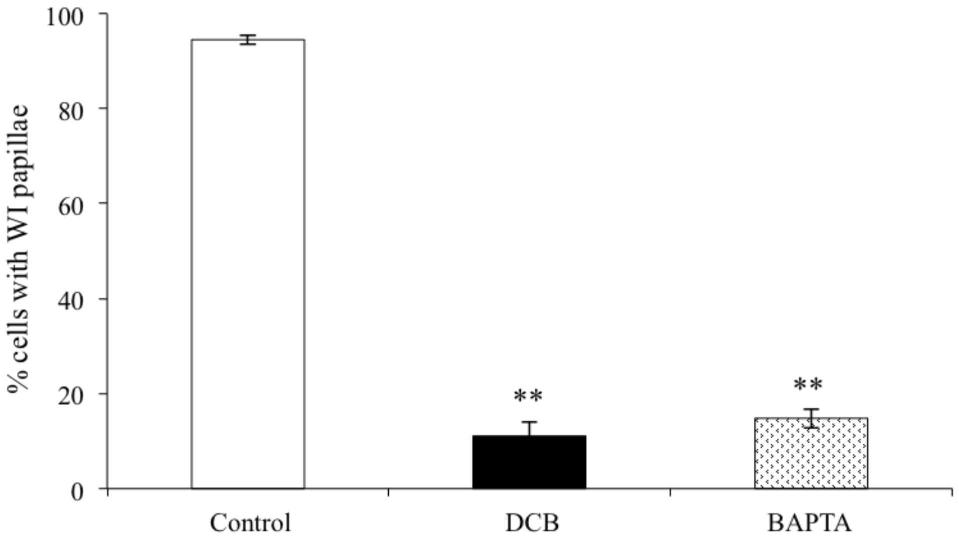

Supplementary Figure S4. Effect of DCB and BAPTA on wall ingrowth (WI) papillae formation in adaxial epidermal cells of cultured *V. faba* cotyledons. Percentages of adaxial epidermal cells with WI papillae in cotyledons cultured for 15 h on MS medium alone (control) or MS medium supplemented with 5 µM DCB or 600 µM BAPTA. Data represent Means ± SEs of six replicate cotyledons for each treatment with 100 cells scored per replicate (n = 6). Asterisk indicates a significant difference from the control (Student's *t* test, **P<0.01) with the percentage data arc sine transformed for analysis.

Supplementary Figure S5. Amino acid sequence alignment of VfCesA3A and VfCesA3B against their available homologues in *Arabidopsis thaliana* (At), *Cajanus cajan* (Cc), *Glycine soja* (Gs) and *Medicago sativa* (Ms). Sequence alignment was performed by the Vector NTI Advanced 11.5.1 (Invitrogen, Darmstadt, Germany) AlignX module with the ClustalOmega algorithm (http://www.ebi.ac.uk/Tools/msa/clustalo/). Bars over the amino acid sequence alignment indicate domain structures of the proteins following the assignments made by Kumar M and Turner S (2015) Plant cellulose synthesis: CesA proteins crossing kingdoms. *Phytochemistry* **112**, 91-99. The N-terminus, zinc finger domain and C- terminus are highlighted in blue, the variable regions (VR) are highlighted in red (solid label for VR 1 and patterned label for VR2), conserved regions (CR) are highlighted in green (solid label for CR 1 and patterned label for CR2) and trans-membrane domains are highlighted in black.

```
                 1                                                                                                  100
AtCesA3    (1)   ---------------------MESEGETAGKPMKNIVPQTCQICSDNVGKTVDGDRFVACDICSFPVCRPCYEYERKDGNQSCPQCKTRYKRLKGSPAIP
CcCesA3    (1)   METNLGLVAGSHISNEFIIIRQDGNFPQREVQLQQLHGKTCQLCGDDIGVNADGEPFVACNECAFPVCKNCYEYERRDGNQVCPQCKTRFKRLKGCARVE
GsCesA3    (1)   ---------------------MESEGEAGAKPVTALGAQVCQICSDGVGKTVDGEPFVACDVCAFPVCRPCYEYERKDGNQSCPQCKTRYKRHKGSPAIL
MsCesA3    (1)   --------------------MMDSEGEAGDKPMKTLGSKVCQICGDNIGSAVNGDPFIACGVCAFPVCRPCYEYERKDGNQSCPQCKTRYNKHKGSPAIL
VfCesA3A   (1)   --------------------MMDSEGESGDKPMKTMGSQVCQICGDNIGSTVDGNPFIACGVCAFPVCRPCYEYERKDGNQSCPQCKTRYNKHKGSPAIL
VfCesA3B   (1)   ---------------------MESEEEVGGKPMAALSAQVCQICAENVGKTFDGEPFIACDFCAFPVCRLCYEYERKDGKQSCPQCKTRYKRHKGSPAII

                 101                                                                                                200
AtCesA3    (80)  GDKDEDGL-ADEGTVEFNYPQK---------EKISERMLGWHLTRGKGEEMGEPQYDKEVSHNHLPRLTSRQDTSGEFSAASPERLSVSSTIAGG----K
CcCesA3    (101) GDEEEDDIDDSENEFDFDGRNK------------------PDSK----MSISSEEQDEETSQEHHALVPSSS-------------------TILS-----
GsCesA3    (80)  GDMEEDGAPAADGASDFNYDSENQNQNQNQKQKISERMLSWQLTYSRGEEVGAPNYDKDVSHNHIPLLTSGQEVSGELSAASPERLSMASPAVGGGKRVH
MsCesA3    (81)  GDREEDGG-ADNDANDFKYNSETQTQK----QKIAERMLSWQMAYGRGEEVDAPNYDKEVSHNHIPRLTGGQETSGELSAASPERMSMASPVNARGKRVH
VfCesA3A   (81)  GDREEDGG-ADNDANDFKYNSETQSQK----QKIAERMLSWQMAYGRGEEVDAPNYDKEVSHNHVPRLTGGQETSGELSAASPERMSMASPGNLRGKRVH
VfCesA3B   (80)  GDSEVDGG-ADDGVSDFNYESENQNQN----QKISDRMLGWQLTLGRSEEVGVPNYDKDVSHNHIPRLTNGQEVSGEFSAASPERLSMSSPVVGGGKRVL

                 201                                                                                                300
AtCesA3    (166) -RLPYSSDVNQSPNRRIVDPVGLGNVAWKERVDGWKMKQE-KNTGPVSTQAAS-ERGGVDIDASTDILADEALLNDEARQPLSRKVSIPSSRINPYRMVI
CcCesA3    (155) -KEIISLQARPMDPSKDLAAYGYGSIAWKERMEIWKQRQMKLSNMKKENDSEDSNNVVVGDDDTEFPVMDE-----GR-QPLSRKLHVPSSKINPYRMII
GsCesA3    (180) NIP-YSSDINQSPN-IRAGDPGLGNVAWKERVDGWKMKQE-KNVVPMSTGLAASERGAGDVDASTDVLVDDSLLNDEARQPLSRKVSIPSSRINPYRMVI
MsCesA3    (176) NHPSYSSDLNQSPN-IRVAEPGLGNVAWKERVDGWKMKHDNKNTAPMSTGQATSERGIGDIDASTDVLFDDSLLNDEARQPLSRKVSIPSSRINPYRMVI
VfCesA3A   (176) NHLSYSSDLNQSPN-IRVVEPGLGNVAWKERVDGWKMKHDNKNAIPMSTGQATSERGIGDIDASTDVFFDDSLLNDEARQPLSRKVSIPSSRINPYRMVI
VfCesA3B   (175) NIP-YSSDVNQSPN-MRVVDAGLSNVAGKERVDGWKMKPE-KNVAPMSTGQAASERGVGDIDARSDIFGDESLLNDEARQPLSRKVSIPSSRINPYRMVI
```

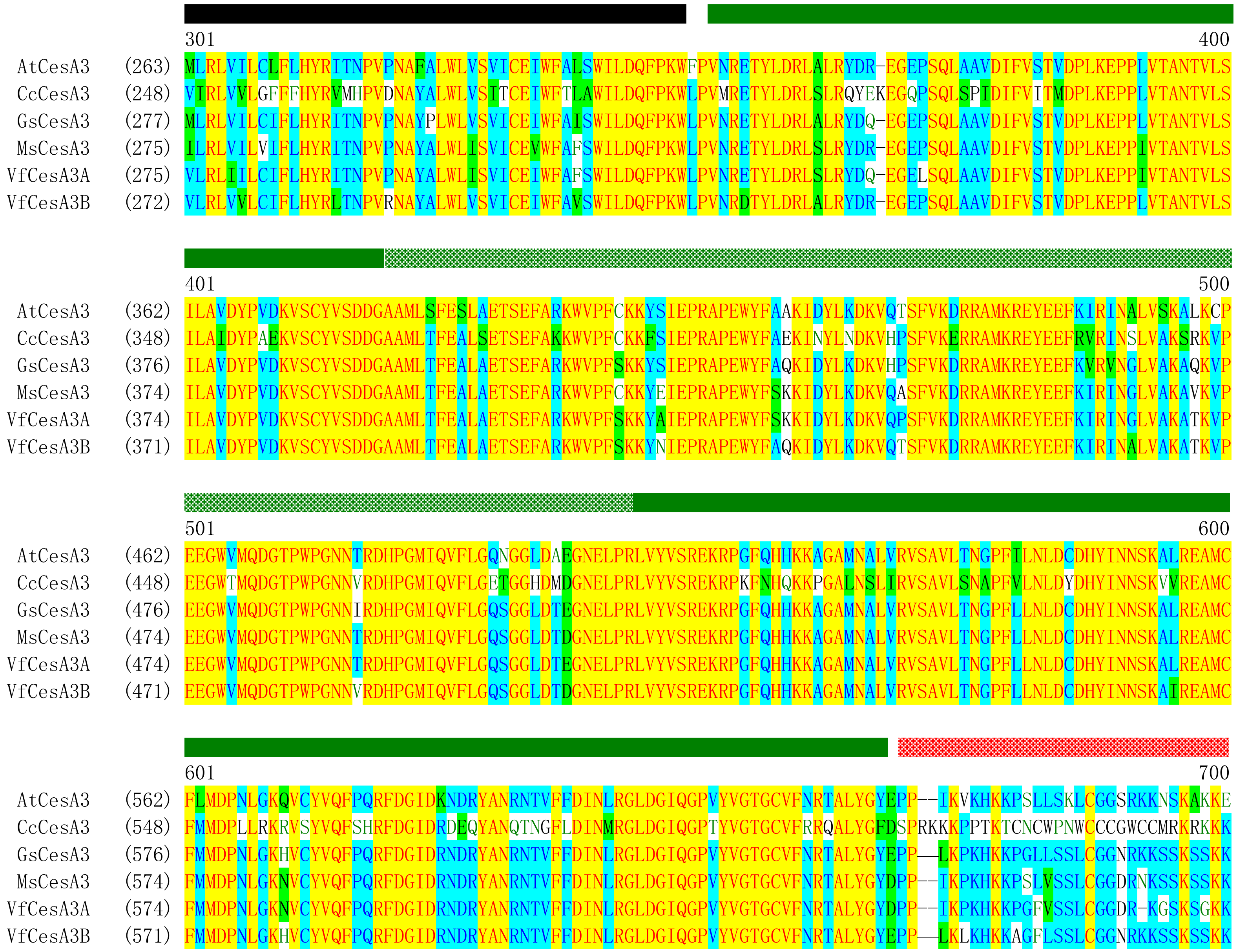

301 400
AtCesA3 (263) MLRLVILCLFLHYRITNPVPNAFALWLVSVICEIWFALSWILDQFPKWFPVNRETYLDRLALRYDR-EGEPSQLAAVDIFVSTVDPLKEPPLVTANTVLS
CcCesA3 (248) VIRLVVLGFFFHYRVMHPVDNAYALWLVSITCEIWFTLAWILDQFPKWLPVMRETYLDRLSLRQYEKEGQPSQLSPIDIFVITMDPLKEPPLVTANTVLS
GsCesA3 (277) MLRLVILCIFLHYRITNPVPNAYPLWLVSVICEIWFAISWILDQFPKWLPVNRETYLDRLALRYDQ-EGEPSQLAAVDIFVSTVDPLKEPPLVTANTVLS
MsCesA3 (275) ILRLVILVIFLHYRITNPVPNAYALWLISVICEVWFAFSWILDQFPKWLPVNRETYLDRLSLRYDR-EGEPSQLAAVDIFVSTVDPLKEPPIVTANTVLS
VfCesA3A (275) VLRLIILCIFLHYRITNPVPNAYALWLISVICEIWFAFSWILDQFPKWLPVNRETYLDRLSLRYDQ-EGELSQLAAVDIFVSTVDPLKEPPIVTANTVLS
VfCesA3B (272) VLRLVVLCIFLHYRLTNPVRNAYALWLVSVICEIWFAVSWILDQFPKWLPVNRDTYLDRLALRYDR-EGEPSQLAAVDIFVSTVDPLKEPPLVTANTVLS
401 500
AtCesA3 (362) ILAVDYPVDKVSCYVSDDGAAMLSFESLAETSEFARKWVPFCKKYSIEPRAPEWYFAAKIDYLKDKVQTSFVKDRRAMKREYEEFKIRINALVSKALKCP
CcCesA3 (348) ILAIDYPAEKVSCYVSDDGAAMLTFEALSETSEFAKKWVPFCKKFSIEPRAPEWYFAEKINYLNDKVHPSFVKERRAMKREYEEFRVRINSLVAKSRKVP
GsCesA3 (376) ILAVDYPVDKVSCYVSDDGAAMLTFEALAETSEFARKWVPFSKKYSIEPRAPEWYFAQKIDYLKDKVHPSFVKDRRAMKREYEEFKVRVNGLVAKAQKVP
MsCesA3 (374) ILAVDYPVDKVSCYVSDDGAAMLTFEALAETSEFARKWVPFCKKYEIEPRAPEWYFSKKIDYLKDKVQASFVKDRRAMKREYEEFKIRINGLVAKAVKVP
VfCesA3A (374) ILAVDYPVDKVSCYVSDDGAAMLTFEALAETSEFARKWVPFSKKYAIEPRAPEWYFSKKIDYLKDKVQPSFVKDRRAMKREYEEFKIRINGLVAKATKVP
VfCesA3B (371) ILAVDYPVDKVSCYVSDDGAAMLTFEALAETSEFARKWVPFSKKYNIEPRAPEWYFAQKIDYLKDKVQTSFVKDRRAMKREYEEFKIRINALVAKATKVP
501 600
AtCesA3 (462) EEGWVMQDGTPWPGNNTRDHPGMIQVFLGQNGGLDAEGNELPRLVYVSREKRPGFQHHKKAGAMNALVRVSAVLTNGPFILNLDCDHYINNSKALREAMC
CcCesA3 (448) EEGWTMQDGTPWPGNNVRDHPGMIQVFLGETGGHDMDGNELPRLVYVSREKRPKFNHQKKPGALNSLIRVSAVLSNAPFVLNLDYDHYINNSKVVREAMC
GsCesA3 (476) EEGWVMQDGTPWPGNNIRDHPGMIQVFLGQSGGLDTEGNELPRLVYVSREKRPGFQHHKKAGAMNALVRVSAVLTNGPFLLNLDCDHYINNSKALREAMC
MsCesA3 (474) EEGWVMQDGTPWPGNNTRDHPGMIQVFLGQSGGLDTDGNELPRLVYVSREKRPGFQHHKKAGAMNALVRVSAVLTNGPFLLNLDCDHYINNSKALREAMC
VfCesA3A (474) EEGWVMQDGTPWPGNNTRDHPGMIQVFLGQSGGLDTEGNELPRLVYVSREKRPGFQHHKKAGAMNALVRVSAVLTNGPFLLNLDCDHYINNSKALREAMC
VfCesA3B (471) EEGWVMQDGTPWPGNNVRDHPGMIQVFLGQSGGLDTDGNELPRLVYVSREKRPGFQHHKKAGAMNALVRVSAVLTNGPFLLNLDCDHYINNSKAIREAMC
601 700
AtCesA3 (562) FLMDPNLGKQVCYVQFPQRFDGIDKNDRYANRNTVFFDINLRGLDGIQGPVYVGTGCVFNRTALYGYEPP--IKVKHKKPSLLSKLCGGSRKKNSKAKKE
CcCesA3 (548) FMMDPLLRKRVSYVQFSHRFDGIDRDEQYANQTNGFLDINMRGLDGIQGPTYVGTGCVFRRQALYGFDSPRKKKPPTKTCNCWPNWCCCGWCCMRKRKKK
GsCesA3 (576) FMMDPNLGKHVCYVQFPQRFDGIDRNDRYANRNTVFFDINLRGLDGIQGPVYVGTGCVFNRTALYGYEPP—LKPKHKKPGLLSSLCGGNRKKSSKSSKK
MsCesA3 (574) FMMDPNLGKNVCYVQFPQRFDGIDRNDRYANRNTVFFDINLRGLDGIQGPVYVGTGCVFNRTALYGYDPP--IKPKHKKPSLVSSLCGGDRNKSSKSSKK
VfCesA3A (574) FMMDPNLGKNVCYVQFPQRFDGIDRNDRYANRNTVFFDINLRGLDGIQGPVYVGTGCVFNRTALYGYDPP--IKPKHKKPGFVSSLCGGDR-KGSKSGKK
VfCesA3B (571) FMMDPNLGKHVCYVQFPQRFDGIDRNDRYANRNTVFFDINLRGLDGIQGPVYVGTGCVFNRTALYGYEPP—LKLKHKKAGFLSSLCGGNRKKSSKSSKK

```
                701                                                                                           800
AtCesA3   (660) SDKK-KSGRHTDSTVPVFNLDDIEEGVEG-----------AGFDDEKALLMSQMSLEKRFGQSAVFVASTLMENGGVPPSATPENLLKEAIHVISCGYED
CcCesA3   (648) KLKKPKFEIMDSSHRKVHSEASLVEGALQYIELS-----SKGTEDEFSAHISNQKFVKKFGQSPIFIASTQLVDGETLKHGNLASQLTEAIHVISCGYEE
GsCesA3   (674) GTDKKKSNKHVDPTVPIFNLEDIEEGVEGTPLLLIHMFLGTGFDDEKSLLMSQMSLEKRFGQSAVFVASTLMENGGVPQSATPETLLKEAIHVISCGYED
MsCesA3   (672) DSKKNKSSKHVDPTVPVFSLEDIEEGVEG-----------AGFDDEKSLLMSQMSLERRFGQSAVFVASTLMENGGVPQSATPETLLKEAIHVISCGYED
VfCesA3A  (671) GSKK-KSSKHVDPTVPIFSLEDIEEGVEG-----------AGFDDEKSLLMSQMSLEKRFGQSAVFVASTLMENGGVPQSATPETLLKEAIHVISCGYED
VfCesA3B  (669) GSDKKKSSKHVDSTVPIFSLEDIEEGVE-----------GSGFDDEKTLLMSQVSLEKRFGQSAVFVASTLMENGGVPQSATPETLLKEAIHVISCGYED
```

```
                801                                                                                           900
AtCesA3   (748) KSDWGMEIGWIYGSVTEDILTGFKMHARGWRSIYCMPKLPAFKGSAPINLSDRLNQVLRWALGSVEILFSRHCPIWYGYNGRLKFLERFAYVNTTIYPIT
CcCesA3   (743) KTEWGKEVGWIYGSVTEDILTGFKMHCHGWRSIYCIPKRTGFKVSTPRHLSNGLQQVFQWALGSTEIFMSKHCPLWYGYGGGLKWLQRISYINAIVYPLT
GsCesA3   (774) KTDWGSEIGWIYGSVTEDILTGFKMHARGWRSIYCMPKRPAFKGSAPINLSDRLNQVLRWALGSVEILFSRHCPIWYGYGGRLKWLERFAYVNTTIYPVT
MsCesA3   (761) KSEWGTEIGWIYGSVTEDILTGFKMHARGWRSIYCMPKLAAFKGSAPINLSDRLNQVLRWALGSVEILLSRHCPIWYGYSGRLKWLERFAYINTTIYPIT
VfCesA3A  (759) KSEWGTEIGWIYGSVTEDILTGFKMHARGWRSIYCMPKLAAFKGSAPINLSDRLNQVLRWALGSVEILLSRHCPIWYGYSGRLKWLERFAYINTTIYPIT
VfCesA3B  (758) KTEWGTEIGWIYGSVTEDILTGFKMHARGWRSIYCMPKRPAFKGSAPINLSDRLNQVLRWALGSVEILFSRHCPIWYGYGGRLKWLERLAYINTTIYPVT
```

```
                901                                                                                          1000
AtCesA3   (848) SIPLLMYCTLPAVCLFTNQFIIPQISNIASIWFLSLFLSIFATGILEMRWSGVGIDEWWRNEQFWVIGGVSAHLFAVFQGILKVLAGIDTNFTVTSKASD
CcCesA3   (843) SIPLVVYCTLPAVCLLTGKFIIPELSNTAGMWFIAVFFCIFTTSVLEMRWSGVTVDEWWRNEQFWVIGGVSAHFLALFLGIFKVLAGVNTNFIVTSKVDD
GsCesA3   (874) AIPLLIYCILPAVCLLTNKFIIPQISNLASIWFISLFLSIFATGILEMRWSGVGIDEWWRNEQFWVIGGVSAHLFAVFQGLLKVLAGIDTNFTVTSKASD
MsCesA3   (861) SIPLLMYCTLPAVCLLTNKFIIPQISNIASIWFISLFLSIFATGILEMRWSGVGIDEWWRNEQFWVIGGVSAHLFAVFQGLLKVLAGIDTNFTVTSKASD
VfCesA3A  (859) SIPLLMYCTLPAVCLLTNKFIIPQISNIASIWFISLFLSIFATGILEMRWSGVGIDEWWRNEQFWVIGGVSAHLFAVFQGLLKVLAGIDTNFTVTSKASD
VfCesA3B  (858) AIPLLLYCTLPAVCLLTNKFIIPQISNLASIWFISLFLSIFATGILEMRWSGVGIDEWWRDEQFWVIGGVSAHLFAVFQGLLKVLAGIDTNFTVTSKASD
```

```
                1001                                                                                         1100
AtCesA3   (948) EDGDFAELYLFKWTTLLIPPTTLLIVNLVGVVAGVSYAINSGYQSWGPLFGKLFFAFWVIVHLYPFLKGLMGRQNRTPTIVVVWSVLLASIFSLLWVRID
CcCesA3   (943) DK-EHSDMFGLKWTTLLIIPTTLLILNIIAVVAGVSYAINNGFVSWGPLFGKLLFSLWVILHLYPFLKGMMGRHNRTPTIVLVWAILLASFFSVLWVKID
GsCesA3   (974) EDGDFAELYMFKWTTLLIPPTTLLIINMIGVVAGISYAINSGYQSWGPLFGKLFFAFWVIIHLYPFLKGLMGRQNRTPTIVVVWSILLASIFSLLWVRID
MsCesA3   (961) EDGDSAELYMFKWTTLLIPPTTLLIINLVGVVAGISYAVNSGYQSWGPLFGKLFFAFWVIIHLYPFLKGLMGRQNRTPTIVVVWSILLASIFSLLWVRID
VfCesA3A  (959) EDGDSAELYMFKWTTLLIPPTTLLIINLVGVVAGISYAVNSGYQSWGPLFGKLFFAFWVIIHLYPFLKGLMGRQNRTPTIVVVWSILLASIFSLLWVRID
VfCesA3B  (958) EDGDSAELYMFKWTTLLIPPTTLLIINLVGVVAGISYAINSGYQSWGPLFGKLFFAFWVIVHLYPFLKGLMGRQNRTPTIVVVWSILLASIFSLLWVRVD
```

```
                  1101              1119
AtCesA3   (1048)  PFTSRVTGPDILECGINC-
CcCesA3   (1042)  PFLPKSDGPILEECGLDCN
GsCesA3   (1074)  PFTTRVTGPDVEECGINC-
MsCesA3   (1061)  PFTTRVTGPKSEMCGINC-
VfCesA3A  (1059)  PFTTRVTGPDSQMCGINC-
VfCesA3B  (1058)  PFTTRVTGPKAEVCGINC-
```

Supplementary Figure S6. Nucleotide sequence alignment of *VfCesA3A* and *VfCesA3B*. Sequence alignment was performed by the Vector NTI Advanced 11.5.1 (Invitrogen, Darmstadt, Germany) AlignX module with the ClustalOmega algorithm (http://www.ebi.ac.uk/Tools/msa/clustalo/). Note differences in nucleotides between the two sequences occur randomly throughout the coding region.

```
                   1                                                                                                  100
VfCesA3A     (1)   ATGATGGACTCAGAAGGGAATCTGGGGATAAGCCGATGAAGACGATGGGTAGCCAAGTCTGCCAGATATGTGGTGATAATATTGGTAGCACTGTTGATG
VfCesA3B     (1)   ---ATGGAGTCAGAAGAGGAAGTTGGGGGGAAACCAATGGCAGCATTGAGTGCTCAAGTATGCCAGATTTGTGCAGAAAATGTTGGGAAGACTTTTGATG
                   101                                                                                                200
VfCesA3A   (101)   GGAATCCGTTCATTGCTTGCGGTGTTTGTGCCTTCCCTGTCTGTAGGCCGTGTTATGAGTATGAAAGGAAAGATGGGAATCAGTCTTGCCCCCAGTGCAA
VfCesA3B    (98)   GCGAACCTTTCATTGCATGCGATTTTTGTGCTTTTCCTGTTTGTAGACTTTGCTATGAGTATGAAAGGAAGGACGGGAAACAGTCTTGTCCCCAGTGTAA
                   201                                                                                                300
VfCesA3A   (201)   AACTCGGTACAATAAGCACAAAGGTAGTCCTGCAATTCTTGGAGACCGGGAAGAGGATGGTGGTGCTGATAATGATGCCAATGACTTCAAGTACAACTCA
VfCesA3B   (198)   AACCCGATACAAGAGGCATAAAGGAAGTCCTGCGATAATTGGAGACAGCGAAGTGGATGGAGGTGCTGATGATGGTGTTAGTGACTTCAATTATGAATCG
                   301                                                                                                400
VfCesA3A   (301)   GAAACTCAGAGCCAAAAGCAAAAGATTGCAGAACGCATGTTGAGCTGGCAAATGGCTTATGGCCGAGGTGAGGAGGTCGATGCTCCAAATTATGATAAGG
VfCesA3B   (298)   GAAAATCAAAACCAAAACCAGAAGATTTCAGATCGCATGTTGGGGTGGCAATTGACACTTGGGCGATCAGAAGAAGTTGGCGTTCCAAATTATGATAAGG
                   401                                                                                                500
VfCesA3A   (401)   AAGTTTCTCACAATCACGTTCCTCGGCTAACCGGCGGACAAGAGACATCTGGAGAATTGTCTGCAGCCTCACCTGAGAGGATGTCAATGGCATCTCCTGG
VfCesA3B   (398)   ATGTTTCTCACAATCACATTCCGCGACTGACAAATGGACAAGAGGTGTCTGGAGAGTTTTCTGCAGCCTCACCTGAGAGGCTATCAATGTCATCTCCCGT
                   501                                                                                                600
VfCesA3A   (501)   AAATCTTCGTGGGAAACGAGTTCATAATCATCTATCATATTCGTCCGATCTTAATCAATCACCGAATATCAGGGTTGTTGAACCAGGATTGGGAAATGTA
VfCesA3B   (498)   AGTTGGTGGGGGAAGCGCGTCCTCAATATTC---CATATTCATCTGATGTTAATCAATCTCCAAATATGAGGGTTGTGGACGCAGGATTAAGCAATGTA
                   601                                                                                                700
VfCesA3A   (601)   GCATGGAAAGAAAGAGTTGATGGGTGGAAAATGAAGCACGATAATAAGAACGCTATTCCAATGAGCACTGGTCAAGCTACATCTGAAAGAGGAATCGGAG
VfCesA3B   (595)   GCGGGGAAAGAAAGAGTTGATGGGTGGAAGATGAAGCCCGAA---AAGAATGTTGCTCCAATGAGCACAGGCCAAGCAGCTTCTGAAAGAGGGGTTGGAG
                   701                                                                                                800
VfCesA3A   (701)   ATATTGATGCCAGTACTGATGTGTTTTTCGATGATTCCTTGTTGAATGATGAAGCTCGGCAACCTCTTTCGAGGAAGGTTTCTATTCCATCTTCCAGAAT
VfCesA3B   (692)   ATATTGATGCCAGATCTGATATATTTGGCGATGAATCATTGTTGAATGATGAAGCTCGACAACCTCTTTCCAGGAAGGTTTCAATTCCGTCATCTAGAAT
                   801                                                                                                900
VfCesA3A   (801)   AAATCCATACCGTATGGTCATTGTTCTGCGGCTTATTATCCTCTGCATTTTCTTGCACTACCGAATTACAAATCCTGTACCCAATGCGTATGCATTGTGG
VfCesA3B   (792)   AAATCCGTATCGCATGGTCATTGTTTTGCGACTTGTTGTTCTTTGCATTTTCTTGCATTATCGATTAACAAATCCGGTGCGAAATGCATATGCTTTATGG
                   901                                                                                               1000
VfCesA3A   (901)   TTAATATCAGTTATATGTGAAATTTGGTTTGCCTTTTCTTGGATATTGGATCAGTTCCCCAAATGGCTTCCTGTGAACCGTGAAACATATCTTGACAGGC
VfCesA3B   (892)   TTGGTATCAGTTATATGTGAGATTTGGTTTGCCGTCTCATGGATTTTGGATCAATTCCCTAAGTGGCTTCCTGTCAATCGCGACACATATCTCGACAGAC
                   1001                                                                                              1100
VfCesA3A  (1001)   TTTCATTAAGATATGATCAGGAAGGGGAACTGTCACAGCTAGCAGCAGTTGACATTTTCGTCAGTACTGTTGATCCCTTAAAGGAGCCCCCAATTGTGAC
VfCesA3B   (992)   TCGCTCTGAGATATGACCGGGAAGGAGAACCGTCTCAACTAGCAGCTGTTGACATTTTTGTCAGCACTGTTGACCCGTTAAAGGAACCTCCACTTGTGAC
                   1101                                                                                              1200
VfCesA3A  (1101)   AGCCAATACTGTACTCTCAATTCTTGCTGTTGACTACCCAGTAGACAAGGTCTCCTGTTATGTCTCTGATGATGGTGCTGCTATGTTGACATTTGAGGCT
VfCesA3B  (1092)   TGCGAATACCGTACTTTCTATTCTCGCAGTTGACTATCCAGTGGATAAGGTCTCCTGCTACGTCTCTGACGATGGTGCCGCGATGTTGACATTTGAAGCT
                   1201                                                                                              1300
VfCesA3A  (1201)   CTTGCCGAGACATCAGAATTCGCTAGGAAATGGGTCCCTTTCAGTAAGAAATATGCAATTGAACCGCGAGCTCCTGAGTGGTACTTTTCAAAGAAAATTG
VfCesA3B  (1192)   CTTGCTGAGACGTCTGAGTTTGCAAGAAAATGGGTTCCTTTCAGCAAGAAATATAATATTGAACCCCGTGCACCTGAGTGGTACTTTGCGCAGAAGATTG
                   1301                                                                                              1400
VfCesA3A  (1301)   ACTATTTGAAAGATAAGGTCCAACCTTCGTTCGTCAAAGATCGTAGAGCAATGAAGAGAGAATATGAAGAATTTAAAATTCGTATCAATGGACTTGTTGC
VfCesA3B  (1292)   ACTACTTGAAAGATAAGGTTCAGACATCGTTTGTCAAGGATCGTAGAGCAATGAAGAGAGAGTACGAAGAATTTAAAATCCGTATTAATGCACTTGTTGC
```

```
                    1401                                                                                               1500
VfCesA3A  (1401)    AAAAGCAACGAAAGTTCCTGAAGAAGGATGGGTGATGCAAGATGGTACACCTTGGCCTGGAAACAACACCAGAGACCATCCAGGAATGATCCAGGTTTTC
VfCesA3B  (1392)    AAAGGCAACGAAAGTTCCGGAAGAAGGGTGGGTAATGCAAGATGGAACGCCGTGGCCTGGAAATAATGTAAGAGATCATCCGGGAATGATTCAGGTTTTC
                    1501                                                                                               1600
VfCesA3A  (1501)    TTGGGCCAAAGTGGAGGACTTGATACTGAGGGTAATGAACTTCCGCGTTTAGTCTATGTTTCTCGTGAAAAGCGTCCAGGGTTCCAACATCACAAGAAGG
VfCesA3B  (1492)    TTAGGCCAAAGTGGAGGACTTGATACTGACGGTAATGAACTTCCACGTTTAGTCTATGTTTCTCGTGAAAAGCGTCCAGGTTTCCAACATCACAAGAAAG
                    1601                                                                                               1700
VfCesA3A  (1601)    CTGGTGCCATGAATGCACTTGTTCGAGTATCAGCTGTCCTTACCAATGGACCTTTCTTATTGAATCTTGATTGTGATCATTACATAAACAACAGCAAGGC
VfCesA3B  (1592)    CCGGTGCCATGAATGCGCTTGTTCGAGTATCTGCCGTCCTTACGAATGGACCTTTCTTATTGAATCTTGATTGCGATCACTATATAAACAACAGCAAGGC
                    1701                                                                                               1800
VfCesA3A  (1701)    CTTGAGGGAAGCTATGTGTTTTATGATGGATCCCAACCTTGGAAAAATGTTTGCTATGTCCAATTTCCACAGAGGTTTGATGGTATTGATAGAAATGAT
VfCesA3B  (1692)    CATAAGAGAAGCAATGTGCTTTATGATGGATCCTAACCTCGGTAAACATGTTTGCTATGTCCAGTTTCCACAGAGGTTTGATGGAATTGATAGGAACGAT

                    1801                                                                                               1900
VfCesA3A  (1801)    CGATATGCCAATCGTAATACCGTTTTCTTTGACATTAACTTGCGAGGATTGGATGGCATTCAAGGCCCAGTTTATGTGGGTACTGGATGTGTCTTCAATA
VfCesA3B  (1792)    CGATATGCTAATCGTAATACTGTTTTCTTTGACATAAACTTGAGAGGTTTGGATGGCATTCAAGGTCCTGTTTATGTCGGTACTGGATGCGTCTTTAATA
                    1901                                                                                               2000
VfCesA3A  (1901)    GAACTGCTTTATATGGTTATGATCCTCCTATTAAACCCAAGCATAAAAAGCCTGGATTTGTTTCTTCACTTTGTGGTGGAGATCGAAAGG---GCTCAAA
VfCesA3B  (1892)    GGACAGCTTTATACGGTTACGAACCTCCTCTAAAACTGAAGCATAAAAAGGCCGGGTTTCTATCATCCCTCTGTGGTGGAAATAGAAAGAAGAGTTCAAA
                    2001                                                                                               2100
VfCesA3A  (1998)    ATCTGGCAAGAAAGGCTCAAAAAGAAA---TCTAGCAAGCATGTTGATCCAACTGTGCCCATCTTTAGTCTAGAGGATATAGAAGAAGGGGTGGAAGGT
VfCesA3B  (1992)    GTCTAGTAAGAAAGGATCGGACAAGAAAAAGTCAAGCAAGCATGTCGACTCAACTGTGCCTATCTTCAGTCTGGAGGATATAGAAGAAGGAGTCGAAGGT
                    2101                                                                                               2200
VfCesA3A  (2095)    GCTGGATTTGATGATGAGAAATCCCTTCTCATGTCTCAAATGAGCCTCGAGAAAAGGTTTGGTCAATCTGCTGTTTTTGTTGCATCTACACTTATGGAAA
VfCesA3B  (2092)    AGTGGATTTGATGATGAGAAAACACTACTTATGTCACAAGTGAGCCTTGAGAAAAGATTTGGTCAGTCGGCTGTTTTTGTTGCGTCTACGCTGATGGAAA
                    2201                                                                                               2300
VfCesA3A  (2195)    ATGGCGGCGTTCCTCAGTCTGCAACTCCAGAAACTCTCCTTAAGGAGGCAATTCATGTTATCAGTTGTGGTTACGAAGATAAATCAGAATGGGGAACTGA
VfCesA3B  (2192)    ATGGCGGAGTTCCTCAGTCTGCTACTCCTGAAACTCTTTTAAAGGAAGCTATTCATGTCATCAGCTGTGGTTATGAGGATAAAACCGAATGGGGAACAGA
                    2301                                                                                               2400
VfCesA3A  (2295)    GATTGGATGGATCTATGGTTCTGTCACAGAAGATATTCTTACTGGATTTAAGATGCATGCCCGCGGTTGGAGGTCTATATACTGCATGCCCAAGCTTGCA
VfCesA3B  (2292)    GATAGGATGGATTTACGGTTCGGTCACAGAAGATATTCTAACTGGATTTAAGATGCATGCCCGAGGTTGGCGGTCGATATACTGTATGCCTAAGCGGCCA
                    2401                                                                                               2500
VfCesA3A  (2395)    GCATTTAAAGGTTCAGCTCCCATCAATCTTTCTGATCGTTTGAACCAAGTGCTTCGGTGGGCTTTAGGCTCAGTGGAAATTCTACTAAGTCGACATTGTC
VfCesA3B  (2392)    GCATTTAAAGGTTCTGCTCCTATCAATCTTTCAGATCGTCTGAATCAAGTGCTTCGATGGGCTTTAGGTTCTGTCGAGATTCTTTTCAGCCGACATTGTC
                    2501                                                                                               2600
VfCesA3A  (2495)    CCATCTGGTACGGTTATAGTGGAAGGCTAAAGTGGCTTGAGAGGTTTGCCTATATAAACACCACAATCTATCCAATCACTTCCATTCCCCTCCTCATGTA
VfCesA3B  (2492)    CCATCTGGTACGGTTATGGCGGAAGACTAAAGTGGCTCGAGAGACTTGCCTATATAAACACCACAATCTATCCAGTCACCGCCATTCCGCTTCTCTTGTA
                    2601                                                                                               2700
VfCesA3A  (2595)    TTGTACCTTACCTGCTGTTTGTCTCTTGACTAACAAGTTCATTATTCCACAGATTAGTAACATTGCAAGTATTTGGTTTATATCTCTCTTTCTTTCCATC
VfCesA3B  (2592)    TTGTACATTGCCAGCTGTCTGTCTTCTTACTAACAAGTTTATCATTCCTCAGATTAGTAACTTAGCGAGTATATGGTTTATCTCTCTCTTTCTTTCAATC
                    2701                                                                                               2800
VfCesA3A  (2695)    TTTGCAACCGGTATCCTAGAGATGAGGTGGAGTGGTGTTGGAATCGATGAATGGTGGAGAAACGAACAATTTTGGGTTATCGGTGGTGTTTCAGCTCATC
VfCesA3B  (2692)    TTCGCGACGGGAATCCTCGAGATGAGATGGAGCGGTGTTGGAATAGACGAGTGGTGGAGAGACGAACAGTTTTGGGTTATCGGTGGCGTATCAGCGCATC
                    2801                                                                                               2900
VfCesA3A  (2795)    TTTTCGCCGTGTTCCAAGGTTTACTCAAAGTACTTGCTGGAATTGACACAAACTTCACTGTTACCTCAAAAGCATCCGACGAAGACGGAGACTCTGCAGA
VfCesA3B  (2792)    TTTTTGCCGTATTCCAAGGTCTACTAAAAGTGCTAGCCGGAATCGACACAAACTTCACCGTCACCTCCAAAGCATCAGACGAAGACGGAGACTCTGCGGA
```

```
                   2901                                                                                               3000
VfCesA3A  (2895)  ACTATACATGTTTAAATGGACAACACTTCTCATTCCACCAACGACCCTTCTTATTATAAACCTCGTCGGAGTCGTTGCAGGAATCTCCTACGCTGTTAAC
VfCesA3B  (2892)  ACTCTACATGTTCAAATGGACGACCCTTCTCATTCCTCCAACAACACTTCTCATAATAAACTTGGTAGGAGTTGTTGCCGGTATATCCTACGCTATTAAC
                   3001                                                                                               3100
VfCesA3A  (2995)  AGTGGCTACCAATCATGGGGACCACTCTTTGGTAAACTTTTCTTTGCATTTTGGGTGATCATCCATTTATACCCTTTCCTTAAAGGTCTTATGGGACGCC
VfCesA3B  (2992)  AGTGGCTACCAATCATGGGGTCCTCTATTCGGTAAACTTTTCTTTGCGTTTTGGGTTATAGTTCATCTTTACCCTTTCCTTAAAGGTCTCATGGGTCGCC
                   3101                                                                                               3200
VfCesA3A  (3095)  AGAACCGAACGCCAACCATCGTGGTTGTTTGGTCCATTCTTCTTGCATCCATCTTTTCGCTTTTATGGGTTCGAATCGACCCTTTTACAACACGAGTCAC
VfCesA3B  (3092)  AGAATCGAACACCGACCATTGTTGTGGTCTGGTCGATTCTTTTGGCGTCCATTTTCTCGCTATTATGGGTTCGAGTTGATCCGTTTACTACAAGAGTGAC
                   3201                             3234
VfCesA3A  (3195)  CGGTCCTGATTCTCAGATGTGTGGAATCAACTGC
VfCesA3B  (3192)  TGGTCCTAAAGCTGAGGTGTGTGGAATTAACTGC
```

Supplementary Figure S7. Amino acid sequence alignment of VfCSL-D2 and VfCSL-D3 against their available homologues in *Arabidopsis thaliana* (At), *Cajanus cajan* (Cc), *Glycine soja* (Gs) and *Medicago sativa* (Ms). Sequence alignment was performed by the Vector NTI Advanced 11.5.1 (Invitrogen, Darmstadt, Germany) AlignX module with the ClustalOmega algorithm (http://www.ebi.ac.uk/Tools/msa/clustalo/). Bars over the amino acid sequence alignment indicate domain structures of the proteins following the assignments made by Kumar M and Turner S (2015) Plant cellulose synthesis: CesA proteins crossing kingdoms. *Phytochemistry* **112**, 91-99. The N-terminus, zinc finger domain and C- terminus are highlighted in blue, the variable regions (VR) are highlighted in red (solid label for VR 1 and patterned label for VR2), conserved regions (CR) are highlighted in green (solid label for CR 1 and patterned label for CR2) and trans-membrane domains are highlighted in black.

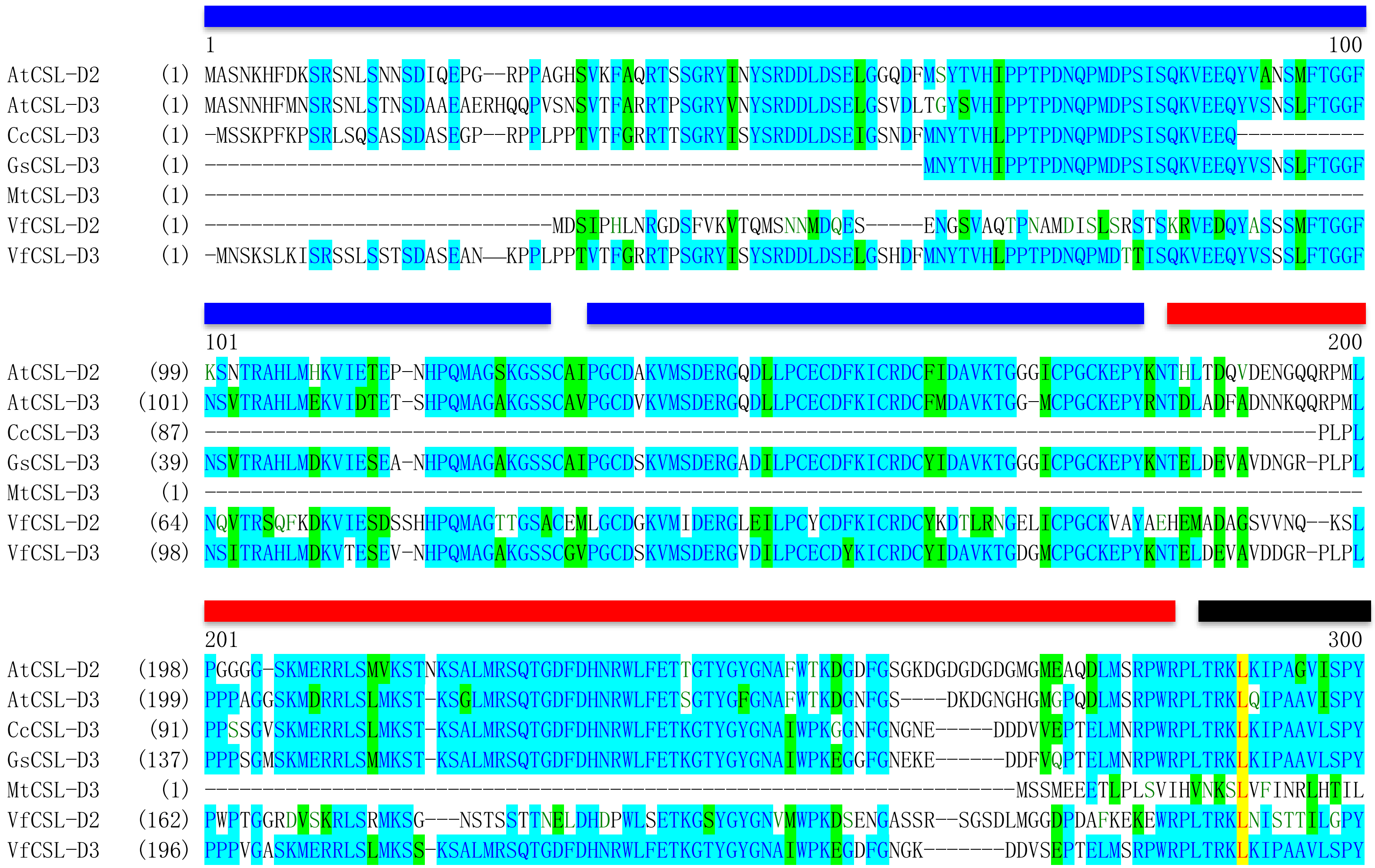

```
                301                                                                                                  400
AtCSL-D2  (297) RLLIFIRIVVLALFLTWRVKHQNPDAVWLWGMSVVCELWFALSWLLDQLPKLCPINRATDLQVLKEKFETPTASNPTGKSDLPGFDVFVSTADPEKEPPL
AtCSL-D3  (294) RLLILIRIVVLALFLMWRIKHKNPDAIWLWGMSVVCELWFALSWLLDQLPKLCPINRATDLNVLKEKFETPTPSNPTGKSDLPGLDMFVSTADPEKEPPL
CcCSL-D3  (185) RLIIFVRMVVLVLFLTWRVKHKNTDAIWLWGMSVVCEIWFAFSWLLDQLPKLCPINRSTDLNVLKEKFEMPSPNNPTGKSDLPGIDVFVSTADPEKEPPL
GsCSL-D3  (230) RLIIFIRLVVLALFLAWRIKHQNSDAVWLWGMSVVCEIWFAFSWLLDQLPKLCPVNRSTDLNVLKEKFETPNPNNPTGKSDLPGIDIFVSTADPEKEPPL
MtCSL-D3   (31) HSIALCFLVYYRLCFFFQDSKTRETPLLPYLLVFSSEIVLSFIWIFDQAFRWNPIKRTVFPERLPENDKLPN------------IDVFICTADPTKEPTL
VfCSL-D2  (257) RCIIFARMVILVLFLRWRVINPNEDAIWLWGMSLVCEIWFAISWLLDQFPKLFPLNRVADLDVLKEKFETPSPHNPTGKSDLPGVDIFVSTADPEKEPPL
VfCSL-D3  (288) RLIIFIRMAALVLFLHWRITHKNTDAIWLWGMSIVCEIWFAFSWLLDQLPKLCPVNRSTDLNVLREKFELPTPENPTGKSDLPGIDVFVSTADPEKEPPL

                401                                                                                                  500
AtCSL-D2  (397) VTANTILSILAAEYPVEKLSCYVSDDGGALLTFEAMAEAASFANIWVPFCRKHAIEPRNPDSYFSLKR-DPYKNKVKSDFVKDRRRVKREFDEFKVRVNS
AtCSL-D3  (394) VTSNTILSILAADYPVEKLACYVSDDGGALLTFEAMAEAASFANMWVPFCRKHNIEPRNPDSYFSLKR-DPYKNKVKADFVKDRRRVKREYDEFKVRINS
CcCSL-D3  (285) VTANTILSILAADYPVEKLSCYVSDDGGALLTFEAMAEAASFANVWVPFCRKHNIEPRNPESYFNLKR-DPYKNKVKPDFVKDRRRVKREYDEFKVRING
GsCSL-D3  (330) VTANTILSILAADYPVEKLSCYVSDDGGALLTFEAMAEAASFANMWVPFCRKHDIEPRNPESYFNLKR-DPYKNKVKPDFVKDRRRVKREYDEFKVRINS
MtCSL-D3  (119) DVMNTVLSAMAMDYPPEKLHVYVSDDGGSPITLNGMKEAWKFAKWWIPFCTRYRISCRCPEAYFSDSQNDGDDFSENVEFIADKRMIKEKYEAFKEGIMR
VfCSL-D2  (357) VTANTILSILAADYPVEKLSCYVSDDGGSLLTFEAMAEATSFANLWVPFCRKHGIEPRNPESYFSLKR-DPYKNKIRSDFVRDRRRVKREYDEFKVRING
VfCSL-D3  (388) VTANTILSILAADYPVEKLSCYVSDDGGALLTFEAMAEAASFANVWVPFCRKHDIEPRNPESYFNLKR-DPYKNKVKPDFVKDRRRLKREYDEFKVRING

                501                                                                                                  600
AtCSL-D2  (496) LPDSIRRRSDAYHAREEIKAMKMQRQNRDDEPMEPVKIPKATWMADGTHWPGTWLTSASDHAKGDHAGIIQVMLKPPSDEPLHG--VSEGFLDLTDVDIR
AtCSL-D3  (493) LPDSIRRRSDAYHAREEIKAMKLQRQNRDEEIVEPVKIPKATWMADGTHWPGTWINSGPDHSRSDHAGIIQVMLKPPSDEPLHG--VSEGFLDLTDVDIR
CcCSL-D3  (384) LPDSIRRRSDAFHAREEIKAMKLQRQNKEYEPLEHVKIPKATWMADGTYWPGTWLNPSPDHSRGDHAGIIQVMLKPPSDEPLLGNADDTKLIDLTDVDIR
GsCSL-D3  (429) LPESIRRRSDAYHAREEIKAMKVQRQNREDDPLETVKIPKATWMADGTHWPGTWLSPTSEHSKGDHAGIIQVMLKPPSDEPLLGSADDTRLIDLTDVDIR
MtCSL-D3  (219) VKEDQN--------------------------------------------------HTTGITGQNHPSTIEVIQENCSG----E---------I--EQVK
VfCSL-D2  (456) LPDSIRRRSDAFNAGEDVKALRLWKEAANDEPIENLKITRTTRMTDGTDWPGTWTTPAPDHSRGDHSSIIQIMSKPPSNEPLIGTASDSNTMDLTEVDIR
VfCSL-D3  (487) LPESIRRRSDAFHAREEIKAMKVVRQNRGDEPVEPIKIPKATWMADGSHWPGTWLNTSSEHSKGDHAGIIQVMLKPPSDEPLIGNADDAKLIDLTDVDIR

                601                                                                                                  700
AtCSL-D2  (594) LPLLVYVSREKRPGYDHNKKAGAMNALVRASAIMSNGPFILNLDCDHYIYNSEALREGMCFMMD-RGGDRLCYVQFPQRFEGIDPSDRYANHNTVFFDVN
AtCSL-D3  (591) LPLLVYVSREKRPGYDHNKKAGAMNALVRASAIMSNGPFILNLDCDHYIYNSQALREGMCFMMD-RGGDRLCYVQFPQRFEGIDPSDRYANHNTVFFDVN
CcCSL-D3  (484) LPLLVYVSREKRPGYDHNKKAGAMNALVRASAIMSNGPFILNLDCDHYIYNSKAMMEGMCFMMD-RGGDRICYVQFPQRFEGIDPSDRYANHNTVFFDVN
GsCSL-D3  (529) LPLLVYVSREKRPGYDHNKKAGAMNALVRASAIMSNGPFILNLDCDHYIYNSKAMREGMCFMMD-RGGDRLCYVQFPQRFEGIDPSDRYANHNTVFFDVN
MtCSL-D3  (254) LPLLVYVSREKKPSHPHHFKAGALNVLYRVSAVISNSPYLLVLDCDMFCGEPASARQAMCFHLDPKKSPSLAFVQFPQKFHNISKNDIYDSQHRSTYTVL
VfCSL-D2  (556) LPMLVYVSREKRPGYDHNKKAGAMNALVRASAVMSNGAFILNLDCDHYIYNSEALREGMCHMMD-RDGERICYVQFPQRFEGIDPSDRYANHNTVFFDVN
VfCSL-D3  (587) LPLLVYVSREKRPGYDHNKKAGAMNALVRASAVMSNGPFILNLDCDHYIYNSKAMREGMCFMMD-RGGDRLCYVQFPQRFEGIDPSDRYANHNTVFFDVN
```

```
                701                                                                                            800
AtCSL-D2  (693) MRALDGLMGPVYVGTGCLFRRIALYGFNPPRSKDFSPSCWSCCFP---RSKKKN-IPEENRALRMS-----DYDDEEMNLSLVPKKFGNSTFLIDSIPVA
AtCSL-D3  (690) MRALDGLMGPVYVGTGCLFRRIALYGFDPPRAKEHHPGFCSCCFS---RKKKKSRVPEENRSLRMGG---DSDDDEEMNLSLVPKKFGNSTFLIDSIPVA
CcCSL-D3  (583) MRALDGLQGPVYVGTGCLFRRVALYGFDPPRSKEHHPGCCSCCFGS-RKKNANANISEENLALRMG-----DSGEEEMNLAMFPKKFGNSSFLIDSIPVA
GsCSL-D3  (628) MRALDGLQGPVYVGTGCLFRRVALYGFDPPRSKEHHTGCCNCCFGRQKKHASLASTPEENRALRMG-----DSDDEEMNLSLFPKKFGNSTFLIDSIPVA
MtCSL-D3  (354) WQGMDGITGPLLSGTGFYMKREALY--------------------------------------------------------------GNYKIKDTDFKLQ
VfCSL-D2  (655) MRALDGLQGPFYVGTGCLFRRTALYGFDPPRLQEEAAEGAGWYGSKKKKSATVASVSEVDSLEDQSLRSGGSIDDEEMNIALIPKKFGNSSLFVESIRVA
VfCSL-D3  (686) MRALDGLQGPVYVGTGCLFRRVALYGFDPPRAKEDHATFCSCCFG--RNKKKLANTSEENRALKMG-----DSDDEEMNLSSFPKKFGNSSFLIDSIPVA

                801                                                                                            900
AtCSL-D2  (784) EFQGRPLADHPAVKNGRPPGALTIPRELLDASTVAEAIAVISCWYEDKTEWGSRIGWIYGSVTEDVVTGYRMHNRGWKSVYCVTKRDAFRGTAPINLTDR
AtCSL-D3  (784) EFQGRPLADHPAVQNGRPPGALTIPRELLDASTVAEAIAVISCWYEDKTEWGSRIGWIYGSVTEDVVTGYRMHNRGWKSVYCVTKRDAFRGTAPINLTDR
CcCSL-D3  (677) EFQGRPLADHPAVKNGRPPGALTIPRELLDASTVAEAISVISCWYEDKTEWGQRVGWIYGSVTEDVVTGYRMHNRGWKSVYCVTKRDAFRGTAPINLTDR
GsCSL-D3  (723) EFQGRPLADHPAVKNGRPPGALTIARDLLDASTVAEAISVISCWYEDKTEWGNRVGWIYGSVTEDVVTGYRMHNRGWKSIYCVTKRDAFRGTAPINLTDR
MtCSL-D3  (392) EYVGTSNEFIKSLKQNCSPNIVTDG----NALPIKETLLLTSCNYEIGTKWGKEVGFMYGTVCEDVHTSIMLSCNGWNSVYCDPPKPQFLGNSATNLNDL
VfCSL-D2  (755) EFQGRPLADHPSVKNGRQPGALILPRDLLNAATVSEAIDVISCWYEDKTEWGDRVGWIYGSVTEDVVTGYRMHNRGWRSVYCVTKRDAFRGSAPINLTDR
VfCSL-D3  (779) EFQGRPLADHPAVKNGRRPGALTIPRELLDASTVAEAISVISCWYEDKTEWGQRVGWIYGSVTEDVVTGYRMHNRGWKSVYCVTKRDAFRGTAPINLTDR

                901                                                                                           1000
AtCSL-D2  (884) LHQVLRWATGSVEIFFSRNN-ALLASSKMKILQRIAYLNVGIYPFTSIFLIVYCFLPALSLFSGQFIVQTLNVTFLVYLLIISITLCLLALLEIKWSGIS
AtCSL-D3  (884) LHQVLRWATGSVEIFFSRNN-AFFASPRMKILQRIAYLNVGIYPFTSFFLIVYCFLPALSLFSGQFIVQTLNVTFLVYLLIISITLCLLALLEIKWSGIS
CcCSL-D3  (777) LHQVLRWATGSVEIFFSRNN-ALLASPRMKFLQRIAYLNVGIYPFTSIFLIVYCFLPALSLFSG------------------------------------
GsCSL-D3  (823) LHQVLRWATGSVEIFFSRNN-ALLASPRMKILQRIAYLNVGIYPFTSIFLIVYCFLPALSLFSGQFIVQTLNVTFLSYLLGITVTLCMLAVLEIKWSGIE
MtCSL-D3  (488) FIQGTRWSSGLLESGLTKVCPLINCPLRMSLLLRFCLTYITCFPLHCLPFWCFAIVPQICLLSGVSLYPKVSEPFFFIYAFIYLSAQTKHLFEALSTGGT
VfCSL-D2  (855) LHQVLRWATGSVEIFFSRNN-GLLANSKLQFLQRVAYLNVAIYPFTSFFLIVYCFLPALSLFTGQFIVQSLQVEFLVYLMGIMVTLIVLAVLEIKWSGIE
VfCSL-D3  (879) LHQVLRWATGSVEIFFSRNN-AIMASTRMKFLQRIAYLNVGIYPFTSFFLIVYCFLPALSLFSGQFIVQNLNVTFLAYLLAITLTLCILAVLEIKWSGIE

                1001                                                                                          1100
AtCSL-D2  (983) LEEWWRNEQFWLIGGTSAHLAAVLQGLLKVVAGVEISFTLTSKSGGDDIDDEFADLYMVKWTS--LMIPPITIIMVNLIAIAVGFSRTIYSVVPQWSKLI
AtCSL-D3  (983) LEEWWRNEQFWLIGGTSAHLAAVIQGLLKVVAGIEISFTLTSKSGGEDVDDEFADLYIVKWTS--LMIPPITIMMVNLIAIAVGFSRTIYSVIPQWSKLI
CcCSL-D3  (840) ----------------HAHLAAVLQGLLKVIAGIEISFTLTSKSGGDDVDDEFADLYIVKWTS--LMIPPITIMMVNLIAIAVGVSRTIYSVIPQWSRLL
GsCSL-D3  (922) LEEWWRNEQFWLIGGTSAHLAAVLQGLLKVVAGIEISFTLTSKSGGDDVDDEFADLYIVKWTS--LMIPPITIMMVNLIAIAVGVSRTIYSVIPQWSRLL
MtCSL-D3  (588) FRTMIIEQRMRMMRSITCHLYGLLDCLMKEFGLREASFMPTNKVKDEEQTMLYQMDKYDFRIPNMFLVPMVALIMINISCFIGGIYRVLS--LGELDKMF
VfCSL-D2  (954) LVDWWRNEQFWMIGGTSAHLVAVLQGLLKVIAGIEISFTLTSKSSG-DDEDDYADLHVIKWSS--LMIPPLTIIMVNIIAVAVAVSKTIYSEDRKWSSLL
VfCSL-D3  (978) LEEWWRNEQFWLIGGTSAHLAAVLQGLLKVVAGIEISFTLTSKSGGDDVDDEFADLYIVKWSS--LMIPPITIMMVNLIAIAVGVSRTIYSTIPQWSRLL
```

```
                      1101                                                                  1170
AtCSL-D2   (1081)     GGVFFSFWVLAHLYPFAKGLMGRRGR---TPTIVYVWSGLVAITISLLWVAINPPAG-NTEIGGNFSFP-
AtCSL-D3   (1081)     GGVFFSFWVLAHLYPFAKGLMGRRGR---TPTIVYVWSGLVAITISLLWVAINPPAG-STQIGGSFTFP-
CcCSL-D3    (922)     GGVFFSFWVLTHLYPFAKGLMGRRGR---TPTIVFVWSGLIAITISLLWVAINPPAG-TNQIGGSFQFP-
GsCSL-D3   (1020)     GGVFFSFWVLAHLYPFAKGLMGRRGR---TPTIVFVWSGLIAITISLLWVAINPPAG-TDQIGGSFQFP-
MtCSL-D3    (686)     IQIYLMAHIILVNYPIIEGIVIRKDKGRISPSVVVTSNVLATILTCALYPLLRKV---------------
VfCSL-D2   (1051)     GGIFFSFWVLSHLYPFAKGLMGKRGK---TPTIVYVWSALLSICISLLWVAINPPSG--NNQIGSFQFP-
VfCSL-D3   (1076)     GGVFFSFWVLAHLYPFAKGLMGRRGK---TPTIVFVWSGLIAIIISLLWVAINPPAGSSNQIGGSFQFP-
```

2.2 Supplementary Tables

Supplementary Table S1. A compilation from literature selected on the basis of clear transmission electron micrographs of the presence/absence of a uniform wall layer in transfer cells across taxonomic groups. The criterion used to determine the presence of a uniform wall layer was that the cell wall underlying wall ingrowth papillae was thicker than in walls free of wall ingrowth papillae. The distinction between the original wall and uniform wall layer was sometimes marked by an electron dense band of wall material (dark band).

Wall labyrinth type	Species	Presence of uniform wall layer (UWL) ± dark band (DB)	Transfer cell type/ location	Reference/ figure(s) number
**Algae**				
Reticulate	*Coleochaete orbicularis*	UWL - DB	Thallus parenchyma/ adjacent to zygote	Graham and Wilcox (1983) Figs 9, 11
**Liverworts**				
Reticulate	*Treubia lacunose*	UWL - DB	Epidermal parenchyma / gametophyte placental region	Carafa et al. (2003) Fig. 2B
**Mosses**				
Reticulate	*Polytrichum commune*	UWL - DB	Parenchyma (deuters)/ leaf bundle	Scheirer (1983) Figs 2 - 4
Reticulate	*Notoligotrichum spp.*	UWL - DB	Epidermal parenchyma /sporophyte placental region	Ligrone and Duckett (2011) Fig. 4a
**Ferns**				
Reticulate	*Regnellidium diphyllum*	UWL - DB	Xylem and phloem parenchyma of frond pinnae	Warmbrodt and Evert (1978) Figs 16, 18
**Gymnosperms**				

Reticulate	*Castanopsis borneensi*	UWL - DB	Cortical cell of root infected by tuberculate mycorrhiza	Haug et al. (1991) Fig. 24
**Angiosperms – Monocots**				
Flange	*Zea mays*	No UWL	Basal endosperm	Monjardino et al. (2013) Fig.3a-c: Rocha et al. (2014) Fig. 1D
Reticulate	*Pappophorum subbulbosum*	UWL + DB	Outer pericarp epidermal/placental pad of seed	Rost et al. (1984) Fig. 14
Reticulate	*Triticum turgidum* var. *durum*	UWL - DB	Nucellar projection/ seed	Wang et al. (1994) Figs 2B, 3D
**Angiosperms – Eudicots**				
Reticulate	*Pisonia grandis*	UWL - DB	Epidermal/ root surrounded by mycorrhizal sheath	Ashford and Allaway (1982) Fig. 3b
Reticulate	*Lactuca*	UWL - DB	Xylem parenchyma/ cotyledonary node	Pate and Gunning (1972) Fig. 1A
Reticulate	*Helianthemum*	UWL - DB	Xylem parenchyma/ node	Jones and Gunning (1976) Fig. 5
Reticulate	*Pisum sativum*	UWL + DB	Companion cells/ leaf minor veins	Wimmers and Turgeon (1991) Fig. 2B
Reticulate	*Pisum arvense*	UWL - DB	Companion cells/ leaf minor veins	Gunning et al. (1968)
Reticulate	*Trifolium alexandrinum*	UWL - DB	Phloem parenchyma/ leaflet petiolule	Winter (1982) Fig. 1b
Reticulate	Soybean	UWL + DB	Central cell of ovule/chalazal end	Folsom and Cass (1986) Fig. 8
Reticulate	Soybean	UWL + DB	Basal cell/ embryo sac	Ditto Fig. 12

Reticulate	*Phoradendron macrophyllum*	UWL - DB	Parenchyma/ adjacent to sinker vessels of mistletoe	Fineran and Calvin (2000) Figs 16, 20
Flange	*Phoradendron macrophyllum*	No UWL	Flange cell	Ditto Figs 20, 23
Reticulate on flange	*Phoradendron macrophyllum*	UWL - DB	Flange walled parenchyma abutting sinker vessels	Ditto Fig. 22
Reticulate	*Duchesnea indica* (host) *Frommeela mexicana* var. *indicae* (fungus)	UWL - DB	Mesophyll infected with fungus	Mims et al. (2001) Fig. 15

Literature Cited

Ashford, A.E. and Allaway, W.G. (1982) A sheathing mycorrhiza on *Pisonia grandis* R. BR. (Nyctaginaceae) with development of transfer cells rather than a hartig net. *New Phytol.* 90**:** 511-519.

Carafa, A., Duckett, J.G. and Ligrone, R. (2003) The placenta in *Monoclea forsteri* Hook. And *Treubia lacunose* (Col.) Prosk: Insights into placental evolution in Liverworts. *Annals Bot.* 92: 299-307.

Fineran, B.A. and Calvin, C.L. (2000) Transfer cells and flange cells in sinkers of the mistletoe *Phoradendron macrophyllum* (viscaceae), and their novel combination. *Protoplasma* 211: 76-93.

Folsom, M.W. and Cass, D.D. (1986) Changes in transfer cell distribution in the ovule of soybean after fertilization. *Can. J. Bot.* 64: 965-972.

Graham, L.E. and Wilcox, L.W. (1983) The occurrence and phylogenetic significance of putative placental transfer cells in the green alga *Coleochaete. Am. J. Bot.* 70: 113-120.

Gunning, B.E.S., Pate, J.S. and Briarty, L.G. (1968) Specialized “transfer cells” in minor veins of leaves and their possible significance in phloem translocation. *J. Cell Biol.* 37: C7-C12.

Haug, I., Weber, R., Oberwinkler, F. and Tschen, J. (1991) Tuberculate mycorrhizas of *Castanopsis borneensis* King and *Engelhardtia roxburghiana* Wall. *New Phytol.* 117: 25-35.

Jones, M.G.K. and Gunning, B.E.S. (1976) Transfer cells and nematode induced giant cells in *Helianthenum. Protoplasma* 87: 273-279.

Ligrone, R. and Duckett, J.G. (2011) Morphology versus molecules in moss phylogeny: New insights (or controversies) from placental and vascular anatomy in *Oedipodium griffithianum. Plant Syst. Evol.* 296: 275-282.

Mims, C.W., Rodriguez-Lother, C. and Richardson, E.A. (2001) Ultrastructure of the host-parasite interaction in leaves of *Duchesnes indica* infected by the rust fungus *Frommeela Mexicana* var. *indicae* as revealed by high pressure freezing. *Can. J. Bot.* 79: 49-57.

Monjardino, P., Rocha, S., Tavares, A.C., Fernandes, R., Sampaio, P., Salema, R., “et al.” (2013) Development of flange and reticulate wall ingrowths in maize (*Zea mays* L.) endosperm transfer cells. *Protoplasma* 250: 495-503.

Pate, J.S. and Gunning, B.E.S. (1972) Transfer cells. *Annu. Rev. Plant Physiol.* **2**3: 173-196.

Rocha, S., Monjardino, P., Mendonca, D., de Camara Machado, A., Fernandes, R., Sampaio, P., "et al." (2014) Lignification of developing maize (*Zea mays* L.) endosperm transfer cells and starchy endosperm cells. *Front. Plant Sci.* 5: 102.

Rost, T.L., De Artucio, P.I. and Risley, E.B. (1984) Transfer cells in the placental pad and caryopsis coat of *Pappophorum subbulbosum* Arech. (Poaceae). *Am. J. Bot.* 71: 948-957.

Scheirer, D.C. (1983) Leaf parenchyma with transfer cell-like characteristics in the moss, *Polytrichum commune* Hedw. *Am. J. Bot.* 70: 987-992.

Wang, H.L., Offler, C.E. and Patrick, J.W. (1994) Nucellar projection transfer cells in the developing wheat grain. *Protoplasma* 182: 39-52.

Wimmers, L.E. and Turgeon, R. (1991) Transfer cells and solute uptake in minor veins of *Pisum sativum* leaves. *Planta* 186: 2-12.

Winter, E. (1982) Salt tolerance of *Trifolium alxandriinum* L. III. Effects of salt on ultrastructure of phloem and xylem transfer cells in petioles and leaves. *Aust. J. Plant Physiol.* 9: 239-250.

Warmbrodt, R.D. and Evert, R.F. (1978) Leaf structure of six species of heterosporous ferns *Botanical Gaz.* 139: 393-429.

Supplementary Table S2. Cotyledon, adaxial epidermal cell and its outer periclinal wall dimensions used to estimate volumes of the outer periclinal original wall and uniform wall layers (see Fig. 1B) of adaxial epidermal cells of *V. faba* cotyledons cultured for specified times. Data are Means ± SEs with 60 cells from four replicate cotyledons for original wall and uniform wall layer measurements (n = 60) and 100 measurements across four replicates for adaxial epidermal cell surface areas and lengths (n = 100).

**Culture time (h)**	**Parameter measured in:**							
	**Cotyledon**	**Adaxial epidermal cell**			**Original wall layer**		**Uniform wall layer**	
	**Adaxial surface area (mm^2)**	**Length (µm)**	**Outer periclinal surface area (μm^2)**	**% cells with uniform wall layer**	**Cross-sectional area (μm^2)**	**Width (µm)**	**Cross-sectional area (μm^2)**	**Width (µm)**
**0**	44.93 ± 0.37	33.67 ± 0.74	575.24 ± 12.39	0 ± 0	20.98 ± 0.98	20.73 ± 1.06	0 ± 0	21.37 ± 1.02
**1**	48.71 ± 0.36	36.63 ± 0.91	602.81 ± 14.62	5.3 ± 2.6	19.81 ± 1.01	19.97 ±0.66	0.21 ± 0.04	22.13 ± 0.24
**2.5**	51.92 ± 0.36	39.37 ± 0.80	636.22 ± 11.21	31.3 ± 4.6	20.06 ± 1.21	18.53 ± 1.19	1.94 ± 0.11	19.55 ± 1.22
**4**	52.68 ± 0.37	40.52 ± 0.69	639.22 ± 10.18	49.2 ± 5.2	20.25 ± 1.27	19.82 ± 0.92	2.95 ± 0.16	20.98 ± 0.90
**6**	52.65 ± 0.33	40.15 ± 0.77	640.04 ± 13.86	79.2 ± 4.7	20.50 ± 1.37	22.98 ± 1.15	4.50 ± 0.28	23.27 ± 1.19
**8**	52.63 ± 0.36	40.29 ± 0.83	641.68 ± 17.46	89.4 ± 2.5	20.35 ± 1.52	21.90 ± 0.90	5.65 ± 0.3	23.81 ± 0.92
**10**	52.85 ± 0.34	40.68 ± 0.85	640.91 ± 16.20	89.7 ± 1.8	20.4 ± 1.21	21.95 ± 0.88	6.60 ± 0.40	23.69 ± 0.86
**12**	52.77 ± 0.34	40.67 ± 0.81	640.97 ± 15.96	91.5 ± 1.7	20.05± 1.64	21.29 ± 1.75	6.75 ± 0.52	22.5 ± 1.25
**15**	52.65 ± 0.48	40.49 ± 0.86	642.53 ± 13.83	89.8 ± 0.7	20.37 ± 1.35	20.51± 1.20	6.93 ± 0.39	20.53 ± 1.12

Supplementary Table S3. Primer sequences used for cloning *VfCesA3B* and for real-time PCR of *VfCesA1, VfCesA3A, VfCesA3B, VfCesA6, VfCS-LD2* and *VfCS-LD3* normalized with *VfEF2α, VfNADHD4, Vf60SL2* and*VfPPlase.*

Primer application	Sequence (5'- 3')
Cloning *VfCesA3B* fragment containing 5'-end by PCR	Forward: CACTTGGGCGATCAGAAGAAG
	Reverse: CTTCGTCTGATGCTTTGGAGG
Cloning *VfCesA3B* full length by 3'-RACE	Forward: TCCAGTCACCGCCATTCCGCTTCTCT
	Reverse: long-CTAATACGACTCACTATAG GGCAAGCAGTGGTATCAACGCAGAGT
	short-CTAATACGACTCACTATAGGGC
Real-time PCR of *VfCesA1*	Forward: TGCTGAGCTTTCTTCGTCTTC
	Reverse: CAACCTTCAACCCTTTCCTTC
Real-time PCR of *VfCesA3A*	Forward: TCAGAAGGGGAATCTGGGGAT
	Reverse: CCATAAGCCATTTGCCAGCTC
Real-time PCR of *VfCesA3B*	Forward: AGCAATGTAGCGGGGAAAGAA
	Reverse: CTGGAAAGAGGTTGTCGAGCT
Rear-time PCR of *VfCesA6*	Forward: ATTTGGCCAGCAACCACATTC
	Reverse: CAGGATTGAGAGGAGGCGATC
Real-time PCR of *VfCSL-D2*	Forward: GGAGGATCAGTATGCGTCGAG
	Reverse: CATCTCACAAGCTGACCCTGT
Real-time PCR of *VfCSL-D3*	Forward: TGCATCTGAAGCAAACAAGCC
	Reverse: TCAGGAGTCGGTGGTAAATGC
Real-time PCR of *VfEF2α*	Forward: GACAACATGATTGAGAGGTCCACC
	Reverse: GGCTCCTTCTCAATCTCCTTACC
Rear-time PCR of *VfNADHD4*	Forward: AGGGTTAGTGAGCACCATGC
	Reverse: ATAGCCAAAGGGAATACGCC
Real-time PCR of *Vf60SL2*	Forward: GATTCTCGATGCAGGGCTAC
	Reverse: CCACTGCTCTTGCTCTTTCC
Real-time PCR of *VfPPlaseG*	Forward: ACCGAAAGAGGAGCAGAACA
	Reverse: GTACTCCGAACTGGAGAGCG

Supplementary Table S4. Effect of culturing *Vicia faba* cotyledons on a MS medium containing 20 µM oryzalin in switching expansion of their adaxial epidermal cells from anisotropic to isotropic growth. Lengths and widths of the adaxial epidermal cells were determined immediately before and following a 4 h culture period on MS medium ± oryzalin. Data are Means ± SEs with 60 cells from four replicate cotyledons (n = 240). Asterisk indicates a significant difference from the control (Student's *t* test, **P<0.01).

Cell dimensions	Cotyledon treatment:		
	Freshly harvested	Cultured for 4 h on:	
		MS medium	Oryzalin/MS medium
Length (µm)	33.37 ± 0.68	40.72 ± 0.64	37.43 ± 0.86 **
Width (µm)	16.06 ± 0.29	16.38 ± 0.27	18.70 ± 0.32 **
Length/width ratio	2.12 ± 0.07	2.55 ± 0.06	2.08 ± 0.07 **

Supplementary Table S5. Temporal profiles of differentially expressed cellulose-related biosynthetic genes in adaxial epidermal and storage parenchyma cells of cultured *V. faba* cotyledons identified from RNA-seq data. The culture interval 0 to 3 h is dominated by uniform wall layer deposition while cell wall deposition at 12 h is exclusively wall ingrowth construction. The RPKM values for *VfCesA3B* and *VfCesA7* are presented as mean values of all unigenes (four for *CesA3B*, two for *CesA7*) verified as separate fragments of *VfCesA3B* and *VfCesA7* by alignment against *CesA3* and *CesA7* sequences of *Arabidopsis thaliana*, *Glycine soja* and *Medicago sativa*. Mean RPKMs ± SEs of six replicate biological samples of adaxial epidermal cells and three replicate biological samples of storage parenchyma cells. Differentially expressed genes were identified as those with a log_2 fold change in expression levels >1 (*- FDR corrected P value < 0.05).

Sequence ID	Name	Relative transcript level (RPKM) in:						Log2 fold change during			
		Adaxial epidermal cells at:			Storage parenchyma cells at:			Adaxial epidermal cells		Storage parenchyma cells	
		0 h	3 h	12 h	0 h	3 h	12 h	0 to 3 h	3 to 12 h	0 to 3 h	3 to 12 h
U22914	[1]*VfCesA1*	48.0 ± 2.1	132.3 ± 1.9	37.5 ± 3.0	29.1 ± 2.4	73.4 ± 4.8	39.9 ± 5.3	1.46*	-1.82	1.34*	-0.88
CL8136.C2	*VfCesA3A*	24.3 ± 1.0	66.3 ± 2.6	18.8 ± 1.3	9.7 ± 1	54 ± 4	47.2± 10.4	1.08*	-1.86	2.48*	-0.19
U23370, U23371, U14683, U18091	*VfCesA3B*	89.1 ± 3.7	60.7 ± 2.0	125.9± 7.0	34.6 ± 3.6	95.9 ± 7.4	60.7 ± 9.8	-0.55	1.05*	1.33*	-0.67
CL1050.C3	*VfCesA6*	87.5 ± 6.8	185.4 ± 9.0	51.0 ± 1.5	28.1 ± 2	84 ± 4	44 ± 5.1	1.45*	-1.82	1.58*	-0.93
U3705, U3706	*VfCesA7*	0.9 ± 0.4	0.1 ± 0.1	0.6 ± 0.1	0.0 ± 0.0	0.1 ± 0.0	0.0 ± 0.0	-3.0	2.4	10	-10
U31756	*VfCesA8*	0.1 ± 0.0	0.0 ± 0.0	0.0 ± 0.0	0.1 ± 0.0	0.1 ± 0.0	0.0 ± 0.0	-1.4	-1.6	0.5	-10
U8857	*VfCSL-D2*	0.6 ± 0.0	3.8 ± 0.4	0.6 ± 0.1	0.0 ± 0.0	0.3 ± 0	0.1 ± 0.0	2.58*	-2.73	4.14	-1.03
CL5957.C1	*VfCSL-D3*	21.5 ± 0.8	59.6 ± 1.5	21.6 ± 1.0	9.5 ± 0.6	18.3 ± 2.6	9.9 ±0.4	1.47*	-1.46	0.95	-0.88
CL1255.C1	*VfCSI1/POM2*	20.9 ± 2.5	42.4 ± 3.1	20.9 ± 1.8	12.4 ± 0.1	48.9 ± 1.6	27.5 ± 6.0	1.02*	-1.02*	1.98*	-0.83
U19135	*VfKOR1*	84.3 ± 2.9	258.5 ± 25.4	95.9 ± 3.6	28.3 ±2.1	138.1 ± 5.1	77.1 ± 5.7	1.62*	-1.43*	2.29*	-0.84
CL2846.C1	*VfCOBRAL1*	19.3 ± 2.1	182.1 ± 5.5	29.2 ± 1.4	11.5 ± 1.3	93.9 ± 3.9	23.7 ± 3.4	3.13*	-2.54*	3.02*	-1.99*
U9631,	*VfCOBRAL2*	83.4 ± 6.1	198.4 ± 4.5	71.4 ± 4.7	9.4 ± 0.7	58.8 ± 2.4	44.2 ± 9.3	1.13*	-1.35*	2.65*	-0.41
U16376	*VfCOBRAL7*	4.4 ± 0.4	23.2 ± 2.5	3.4 ± 0.3	1.8 ± 0.1	6.2 ± 0.6	1.6 ± 0.3	2.40*	-2.78*	1.81*	-1.95*

[1] Amino acid sequences of the presented genes were aligned against their closest Arabidopsis homologue (based on TAIR 10) using cluster Omega (http://www.ebi.ac.uk/Tools/msa/clustalo/). Percentage sequence identity to, and the TAIR ID of, their Arabidopsis homologues are:
U22914: 86% to AtCesA1 (AT4G32410); CL8136.C2: 85% to AtCesA3 (AT5G05170); CesA3B: 83% to AtCesA3 (AT5G05170); CL1050.C3: 83% to AtCesA6 (AT5G64740); CesA7: 87% to AtCesA7 (At5G17420); CesA8: 85% to AtCesA8 (At4G18780); U8857: 69% to AtCSLD2 (AT5G16910); CL5957.C1: 84% to AtCSLD3 (AT3G03050); VfCSI1/POM2: 72% to AtCSI1/POM2 (AT2G22125); VfKOR1: 74% to AtKOR1 (AT5G49720); CL2846.C1: 79% to AtCOBL1 (AT3G02210); U9631: 71% to AtCOBL2 (AT3G29810); U16376: 66% to AtCOBL7 (AT4G16120)
